# Sensitization of the UPR by loss of PPP1R15A promotes fibrosis and senescence in IPF

**DOI:** 10.1038/s41598-021-00769-7

**Published:** 2021-11-03

**Authors:** Susan Monkley, Catherine Overed-Sayer, Helen Parfrey, Doris Rassl, Damian Crowther, Leire Escudero-Ibarz, Nicola Davis, Alan Carruthers, Richard Berks, Marisa Coetzee, Ewa Kolosionek, Maria Karlsson, Leia R. Griffin, Maryam Clausen, Graham Belfield, Cory M. Hogaboam, Lynne A. Murray

**Affiliations:** 1 Translational Science and Experimental Medicine, Research and Early Development, Respiratory and Immunology, BioPharmaceuticals R&D, AstraZeneca, Gothenburg, Sweden; 2 Bioscience COPD/IPF, Research and Early Development, Respiratory and Immunology, BioPharmaceuticals R&D, AstraZeneca, Cambridge, UK; 3 Cambridge Interstitial Lung Disease Service, Royal Papworth Hospital, Cambridge, UK; 4 Royal Papworth Hospital, Cambridge, UK; 5 Neuroscience, BioPharmaceuticals R&D, AstraZeneca, Cambridge, UK; 6 Discovery Sciences, BioPharmaceuticals R&D, AstraZeneca, Cambridge, UK; 7 Biological Services Group, MRC Laboratory of Molecular Biology, Francis Crick Avenue, Cambridge, UK; 8 Babraham Institute, Cambridge, UK; 9 Bioscience COPD/IPF, Research and Early Development, Respiratory and Immunology, BioPharmaceuticals R&D, AstraZeneca, Gothenburg, Sweden; 10 Translational Genomics, Discovery Biology, Discovery Sciences, BioPharmaceuticals R&D, AstraZeneca, Gothenburg, Sweden; 11 Cedars-Sinai Department of Medicine, Los Angeles, CA 90048 USA

**Keywords:** Cell biology, Diseases

## Abstract

The unfolded protein response (UPR) is a direct consequence of cellular endoplasmic reticulum (ER) stress and a key disease driving mechanism in IPF. The resolution of the UPR is directed by PPP1R15A (GADD34) and leads to the restoration of normal ribosomal activity. While the role of PPP1R15A has been explored in lung epithelial cells, the role of this UPR resolving factor has yet to be explored in lung mesenchymal cells. The objective of the current study was to determine the expression and role of PPP1R15A in IPF fibroblasts and in a bleomycin-induced lung fibrosis model. A survey of IPF lung tissue revealed that PPP1R15A expression was markedly reduced. Targeting *PPP1R15A* in primary fibroblasts modulated TGF-β-induced fibroblast to myofibroblast differentiation and exacerbated pulmonary fibrosis in bleomycin-challenged mice. Interestingly, the loss of *PPP1R15A* appeared to promote lung fibroblast senescence. Taken together, our findings demonstrate the major role of PPP1R15A in the regulation of lung mesenchymal cells, and regulation of PPP1R15A may represent a novel therapeutic strategy in IPF.

## Introduction

Idiopathic pulmonary fibrosis (IPF) is an unrelenting, chronic, and progressive lung disease. The rate of decline in lung function varies between patients, with some having a more rapidly progressive disease than others^1,2^. Despite this, all patients will ultimately succumb to respiratory failure. At the cellular level, normal alveolar lung tissue is replaced by excess extracellular matrix as a consequence of aberrant mesenchymal cell proliferation and activation^2^. These mechanisms are often linked to an impaired wound healing response^3^. There are currently two approved drugs for IPF, nintedanib and pirfenidone. Both offer some benefit, albeit patient responsiveness is not predictable or consistent. Furthermore, both drugs show minimal disease modification and have challenging side effect profiles, often limiting their long-term use^4,5^. Therefore, new therapeutic approaches, targeting the underlying cycle of continued injury and impaired repair responses in IPF, are needed.

One process that may perpetuate these aberrant repair processes in IPF is the unfolded protein response (UPR) (see Table S1, Fig. S1 for overview)^6^. The UPR is comprised of three endoplasmic reticulum (ER) stress signalling pathways that are required for maintenance of cellular and ER homeostasis and regulated by the ER resident chaperone HSPA5 (BiP/GPR78)^7^. The UPR is activated in response to ER stress when the capacity for folding secreted and transmembrane proteins is overwhelmed by cellular demand. ER stress has been linked to IPF, specifically as a process of promoting alveolar type 2 epithelial cell apoptosis^8^, and also during fibroblast to myofibroblast differentiation^9^. During periods of ER stress, the UPR restores proteostasis via several mechanisms, most notably the attenuation of protein synthesis by PERK phosphorylation of eIF2α (EIF2A, Table S1). Following resolution of the ER stress, the block on general protein translation is reversed by dephosphorylation of eIF2α, which is directed by PPPIR15A/GADD34 (Table S1, Fig. S1) and leads to the restoration of normal ribosomal activity^10^. *PPPIR15A* is constitutively expressed at low levels but its transcription is rapidly upregulated by ATF4 via DDIT3 (CHOP, Fig. S1) following cell stress, and may enhance ER stress^10,11^. Recently, bleomycin treatment leading to lung fibrosis in mice has been shown to increase ER stress, notably GRP78/BiP, ATF4 and DDIT3 levels^12^. Conversely, the inhibition of ER stress signalling by heterozygous Grp78 gene deletion^13^ or treatment with TUDCA, ameliorated bleomycin-induced lung fibrosis; in the latter case there was a correlated inhibition of PI3K/mTOR pathway activation^12,13^. Interestingly, *Grp78*^−/−^ mice completely lacking this factor are more susceptible to bleomycin-induced fibrosis, with the enhanced susceptibility linked to the loss of AT2 epithelial cells^14^.

Cellular senescence of several cell types including epithelial cells and fibroblasts in the lungs of IPF patients appears to contribute to protein-misfolding stress and to stimulation of the UPR^15^. Indeed, we have shown that the loss or inhibition of DNA damage repair pathways are linked to cellular senescence in IPF and contribute to the progressive nature of disease^16^. Growing evidence suggests that the targeting of senescent cells via senolytic strategies might provide therapeutic benefit in IPF possibly due to the attenuation of UPR^17^.

In this study we focused on the UPR in mouse and human primary lung fibroblasts. We observed that PPPIR15A was reduced in lung fibroblasts in IPF patients and that TGFβ-induced fibroblast activation further decreased *PPPIR15A*. PPPIR15A deficiency or its pharmacological inhibition exacerbated experimental lung fibrosis. Together, this study highlights that PPP1R15A deficiency promotes lung fibroblast senescence and this constitutes a pathological feedforward mechanism that represents an attractive potential target for therapeutic intervention.

## Methods

### Patients

Ethical approval for the use of samples and data collection was obtained (Royal Papworth Hospital Research Tissue Bank (Research Ethics Committee), 08/H0304/56). All methods were carried out in accordance with relevant guidelines and regulations. All patients provided informed consent using the Royal Papworth Hospital Research Tissue Bank consent form. Patients were diagnosed with either sporadic (n = 11) or familial (n = 5) IPF in accordance with ATS/ERS/JRS/ALAT clinical practice guideline^18^. Most patients were male (9/16), age 67.2 ± 7.5 years (mean ± SD, n = 16) and 9 were never smokers. At the time of lung biopsy, forced vital capacity (FVC) was 85.9 ± 11.7% predicted (mean ± SD, n = 13) and gas transfer (TLco) was 53.1 ± 17.7% predicted (mean ± SD, n = 13).

### IPF immunohistochemistry

Formalin fixed, paraffin embedded (FFPE) human lung tissue sections were obtained from patients undergoing diagnostic lung biopsy at Royal Papworth Hospital, Cambridge UK. 5 μm-thick sections of FFPE human lung tissue were subjected to haematoxylin and eosin (H + E)-staining as previously described^19^. For immunohistochemistry, FFPE sections were baked in an oven at 65 °C for 1 h. PT link tanks (Dako, Glostrup, Denmark) were used to perform deparaffinisation and heat-induced epitope retrieval (EnVision FLEX Target Retrieval Solution High pH; Dako). All slides were incubated for 20 min at 97 °C and left in buffer (EnVision FLEX wash buffer; Dako) at room temperature for a minimum of 5 min to cool down. Staining was performed using an automated immunostainer (AutostainerLink48; Dako). The protocol was as follows: slides were incubated for 5 min in an endogenous block (EnVision FLEX peroxidase-blocking reagent; Dako) and then incubated with rabbit polyclonal anti-GADD34 1:100 (10449-1-AP; Proteintech, Chicago, IL, USA) or rabbit monoclonal anti-BiP 1:200 (C50B12; Cell Signaling Technology, Boston, MA, USA)for 30 min. Then all sections were incubated for 30 min in labelled polymer (EnVision FLEX/HRP; Dako). Each individual stage was followed by buffer rinses (EnVision FLEX wash buffer; Dako). Staining was visualised using the chromogen 3,30-diaminobenzidine for 10 min, counterstained with haematoxylin (EnVision FLEX; Dako) for 5 min and manually cover-slipped (Surgipath) with DePeX mounting medium (VWR International, Poole, UK). Semi-quantitative analyses were performed using light microscopy (200 × magnification), where six high power fields were randomly selected from each lung biopsy section and were scored for epithelial staining of GADD34/PPP1R15A and BiP by semi-quantitative analysis by two independent, blinded investigators (HP and DR). Similarly, the H + E stained sections were used to assess for inflammation and fibrosis. Data were analysed by linear regression analysis using Prism software.

### Bleomycin-induced lung fibrosis models

All in vivo mouse experiments were approved by the Institution’s Animal Welfare and Ethical Review Body (AWERB) committee. All in vivo work were conducted under the authority of a UK Home Office issued Project License and in accordance with the Animals (Scientific Procedures) Act 1986. For the bleomycin timecourse analyses, female C57Bl/6 mice received an oropharangeal bleomycin challenge (Blenoxane, Sigma, St. Louis, MO) or sterile saline control as previously described^20^. Mice were then sacrificed at specific timepoints following bleomycin or saline delivery and lungs were collected for analysis. *Ppp1r15a* null mice (*Ppp1r15a*^−/−^) were generated using B6.129P2-PppIr15a/Mmnc sperm (http://www.mmrrc.org) on an C57Bl/6jax background and the colony maintained in the Biological Services Group facility. For the knockout bleomycin studies, age and sex-matched, and wildtype littermate controls received an oropharyngeal bleomycin or saline challenge on Day 0 and were sacrificed on Day 5 or Day 21. For the Sephin1 bleomycin model, mice were challenged as above, and treated daily with either vehicle control (saline, 10 ml/kg) or Sephin1 by oral gavage on Days 14–20 (5 mg/kg). Reporting in this manuscript follows the recommendations of the ARRIVE guidelines.

### Gene expression and protein analyses in mouse fibrosis model

Gene expression levels were quantitated using real time RT-PCR (Applied Biosystems) according to the manufacturer's protocols. Transcript levels of genes of interest measured by quantitative RT-PCR were normalized to housekeeping gene mRNA. For protein analysis, collagen levels were determined in lung homogenates using an established hydroxyproline assay as previously described^21^. TNFα protein levels were measured by MSD analysis (Mesoscale Discovery), according to manufacturer’s protocols.

### Mouse histology

Formalin-fixed and paraffin-embedded lung sections were stained with hematoxylin and eosin to assess gross morphology or Masson’s trichrome stains to visualize collagen deposition.

### In vitro fibroblast assays

IPF lung fibroblasts were used in all experiments (Lonza, cat. no. CC7231; Donor 1: batch no. 8F5049; Donor 2: 8F5060; Donor 3: 8F5061; Donor 4: 0000627840; Donor 5: 6F5002 (5 Donors).

### TGFβ1 activation assay

Fibroblasts were cultured and maintained in DMEM media (Gibco, cat no 31966) with 10% (v/v) FBS (Gibco, cat. no. 12070–106) and 1% (v/v) Penicillin–Streptomycin (Gibco, cat. no. 15140122) at 37 °C in a 5% (v/v) CO_2_ humidified atmosphere. Cells were seeded in 96 well plates at 16,000 cells/well and allowed to adhere for 24 h. Next, cells were washed twice with 100 µl DPBS and starved for 24 h in serum free DMEM. Compound treatment was added using Tecan D300 and normalized with DMSO to 0.1% (v/v) (nintedanib or AZD8055 (mTOR inhibitor) at concentrations 0 µM; 0.1 µM and 10 µM for NGS experiment or at a full dose response for α-SMA staining). Plates were placed in the cell incubator for 1 h, followed by adding DMEM + 0.1%BSA with or without TGFβ1 (final concentration of TGFβ1 in well = 0.123 ng/ml). Plates were incubated for 0 h and 6 h and 24 h (NGS experiment) or 24 h only for α-SMA staining. Plates for NGS experiment were lysed in freshly prepared lysis buffer (60 µl lysis buffer and 3 µl proteinase K) per well. The plates were sealed and incubated at room temperature for 30 min and stored at − 80 °C. Plates for α-SMA staining were fixed with 70 µl of 4% (v/v) formaldehyde (VWR, cat no 9713.1000), incubated at room temperature for 15 min, washed 3 times with DPBS, sealed and stored at 4 °C until staining was performed.

### Immunocytochemistry for αSMA

Cells were permeabilized with 0.2% Tween in PBS for 15 min (100 µl/well). Cells were then blocked with 5% (w/v) BSA in PBS for 1 h. Primary antibody was added (Daco, cat no M0851; 1:250) and incubated for 1 h, followed by washing 3 times with PBS. Next, secondary antibody (Invitrogen, cat no A11001; 1:500) + Hoechst (Invitrogen, cat no H3570; 1:10,000) were added and incubated for 1 h in the dark, followed by washing 3 times with PBS. Plates were sealed and image analysis performed using ImageXpress.

### In vitro senescence assay

IPF fibroblasts were seeded at 5000 cells per well in a 96-well plate. After 24 h incubation at 37 °C and 5% (v/v) CO_2_, the medium was replaced with fresh medium and the cells were treated with the compounds. For the NGS experiment, the mTOR inhibitor, AZD8055, was added at concentrations of 1 µM, 0.01 µM, and 0 µM. For the cell imaging endpoint, AZD8055 or nintedanib were added at ten different concentrations (0–10 µM). All samples were normalised with the vehicle control with 0.1% (v/v) DMSO. After 1 h incubation with the compound, a final concentration of 3 µM etoposide or the vehicle control (0.006% (v/v) DMSO) were added to the cells, after which the cells were incubated for 0, 6 h and, 24 h for the NGS experiment and 72 h for the imaging experiment. The cells for the NGS experiment were harvested by removing the medium and adding 63 µl of freshly prepared lysis buffer (60 µl lysis buffer and 3 µl proteinase K) per well. The plates were sealed and incubated at room temperature for 30 min and stored at − 80 °C. The plates for the imaging endpoint were fixed by addition of 50 µl of 4% (v/v) formaldehyde (VWR, cat. no. 9713.1000) per well. After 15 min incubation, the cells were washed three times with PBS after which they were sealed and stored at 4 °C.

### Senescence assay p21 imaging endpoint

Cells were permeabilised by the addition of 100 µl of 0.1% (v/v) Triton X-100 in PBS per well for 20 min. Thereafter, the cells were blocked by incubation for 1 h with 50 µl blocking buffer (5% (w/v) BSA in PBS) followed by incubation overnight at 4 °C with 50 µl of anti-p21 antibody (Abcam, cat. no. Ab109520) diluted 1:1000 in blocking buffer. The cells were then washed three times for 5 min and incubated with 50 µl of an Alexa Fluor 594-conjugated donkey anti-rabbit secondary antibody (Invitrogen, cat. no. A21207) diluted 1:500 in blocking buffer for 1 h at room temperature in the dark, after which the cells were washed for 5 min with PBS and stained for 15 min with 50 µl Hoechst (Invitrogen, cat. no. H3570) and HCS CellMask™ Deep Red (Life technologies, cat. no. H32721) diluted 1:10,000 and 1:5000 in PBS, respectively. Finally, the cells were washed twice with PBS and imaged using a Yokogawa CV7000 confocal microscope and the images were analysed using the Columbus Image Analysis software (PerkinElmer).

### RNA sequencing

Total RNA was isolated from samples containing a maximum of 50,000 cells/sample using the RNAdvance Cell v2 protocol (Beckman Coulter) in a 96 well plate format on a Beckman Biomek i7 liquid handler instrument (Beckman). The quantity and quality of RNA samples was assessed using the high sensitivity RNA fragment analysis kit on a 96 well Fragment Analyzer (Aligent Technologies). All samples had an RNA integrity number > 9.9 and were deemed of sufficient quality and quality for RNA-seq analysis. Samples were diluted to a final quantity of 50 ng/well of total RNA using a Tecan Fluent liquid handling automation system (Tecan). The KAPA mRNA HyperPrep kit (Roche) was used for reverse transcription, generation of double stranded cDNA and subsequent library preparation and indexing to facilitate multiplexing (IDT), all of which was performed through automation on a Tecan fluent (the manual protocol was adapted for automation in-house). All libraries were quantified with the Fragment Analyzer using the standard sensitivity NGS kit (Aligent Technologies), pooled in equimolar concentrations and quantified with a Qubit Fluorometer (ThermoFisher Scientific) with the DNA HS kit (ThermoFisher Scientific), the library pool was further diluted to 1.9 nM and sequenced at > 20 M paired end reads/sample using the S2 regent kit to 100 cycles on an Illumina Novaseq 6000.

### RNA sequencing data processing

Fastq were processed via the bcbio-nextgen v 1.2.0-b pipeline with Hisat2 (v2.2.0) as the aligner and Salmon (v0.14.2) as the pseudo-aligner. The reference genome was hg38, ensembl annotation release 94. For downstream analysis the TPM values generated from Salmon were used as the expression values. For heatmaps and expression plots log2(TPM) were used unless otherwise stated. Differential expression was performed using DESeq2 (v1.26.0) to generate multiple testing adjusted P-values (represented on plots). Heatmaps were generated using Morpheus (https://software.broadinstitute.org/morpheus) with clustering metric being one minus Pearson correlation and complete linkage. Pathway and expression regulation for UPR genes was extracted from Ingenuity Pathway Analysis (IPA; Qiagen) relationships.

### Re-processing of GSE32537 and GSE47460

Both datasets were reprocessed from raw data obtained from GEO. For GSE32537^22^, the CEL files were processed using RMA method along with the BrainArray (http://brainarray.mbni.med.umich.edu/Brainarray/Database/CustomCDF/genomic_curated_CDF.asp) ensembl gene ID version 24 cdf to collapse the probes to a single gene. This generated normalised expression data on log2 scale with probes collapsed to gene level. Subsequent QC indicated one sample did not correspond to the assigned gender in the metadata and was removed from subsequent tests. Differentially expressed genes were generated by using Limma to compare the IPF samples to the controls controlling for both age and gender as the controls were generally younger than IPF patients. For GSE47460^23^ only the samples on the SurePrint G3 Human GE 8 × 60K Microarray (GPL14450) were used as this the majority of the samples and more genes. The text files obtained from GEO were processed using the Limma package including background correction with the ‘normexp’ method offsetting for internal background measurements, quantile normalization between arrays with log2 transformation and then probes were filtered based on the PA-matrix. Again with this dataset a sample was excluded due to gender mismatch between gene expression and metadata. Additional samples were excluded due to failing QC metrics during processing. Again Limma was used to generate differentially expressed genes comparing the IPF samples to controls controlling for gender and age. To collapse the probes, the probe with the lowest false discovery rate (FDR) for each gene was selected to generate the final data set of differentially expressed genes. The Log2 fold changes and FDR for each of the 2 above data sets was plotted in R to generate the bubble plot.

### Processing of single cell RNA sequencing data to generate differentially expressed genes

#### GSE12296

Data was downloaded from GEO with additional metadata (cell type annotation) provided by authors (A. Misharin) when requested. Data was analysed using Seurat^24^ and only the IPF samples (n = 4) and control samples (N = 10) were analyzed (54,544 cells; 41,170 Control and 13,374 IPF). Differential analysis was performed for each of the annotated cell types provided by the authors comparing IPF to control using the negative binomial method from Seurat(Find Markers).

#### GSE135983

Data was downloaded from GEO as a filtered and processed Seurat object. This object contained data that had been through the QC, normalization and annotation as described by the authors. The object was subsetted to contain only the samples from 10 control and 12 IPF samples (89,326 cells; 31,644 Control, 57,682 IPF). Differential analysis was performed for each of the annotated cell types provided by the authors comparing IPF to control using the negative binomial method from Seurat (Find Markers). HAS1 High fibroblasts had no DE genes, hence were removed from the list containing DGE information.

#### GSE136831

Data was downloaded from GEO as a 78 single datasets (28 normal lung, 32 IPF lung, 18 COPD lung). The metadata describing the cell cluster identity defined by the authors, was included along with addition required metadata The object was subset to contain only the samples from 28 control and 32 IPF samples (243,472 cells). QC and normalization was performed in Seurat. Differential analysis was performed for each of the 37 annotated cell types provided by the authors comparing IPF to control using the negative binomial method from Seurat (Find Markers).

#### GSE128033

Data was downloaded from GEO as 18 single datasets (8 normal lung, 8 IPF lung, 2 bronchioalveolar lavage cell extracts (one fresh, one frozen)). The authors were contacted for the required metadata describing the cell cluster identity defined by the authors (based on Fig. 2b in publication). Using samples for which there was sample barcodes from authors: only data for 4 Control (normal) and 8 IPF samples (47,771 cells). QC and normalization was performed in Seurat. Differential analysis was performed for each of the 23 annotated cell types provided by the authors comparing IPF to control using the negative binomial method from Seurat (Find Markers).

### CRISPR-Cas9 RNP electroporation in primary normal human lung fibroblasts

sgRNA targeting the PPP1R15A gene were purchased from Synthego (Gene Knockout Kit v2 PPP1R15A). The sgRNA sequences are reported in Supplementary Table S1. Lyophilised sgRNA reagents were resuspended in 1 × TE buffer at a concentration of 50 μM. 75 pmol of sgRNA was complexed with 60 pmol of Cas9 protein (10 μg/μl; IDT) (ratio 1.25:1 sgRNA:Cas9) to form ribonucleoprotein (RNP) complexes. The mix was incubated at 37 °C for 15 min. Following complex formation, 60 pmol of electroporator enhancer (100 μM; IDT) was added to the RNP complex and incubated at room temperature for 5 min. Passage 2 cells from primary normal human lung fibroblast donors (NHLF) were electroporated when around 80% confluent. For both the 4D nucleofector and 384 HT nucleofector systems (Lonza), 250,000 cells were electroporated in 20 μl per electroporation condition. In brief, cells were detached and collected by centrifugation (90*g*, 10 min, room temperature) and washed once with phosphate-buffered saline (PBS) solution. Cell pellets containing 250,000 cells per reaction were resuspended in 16.9 μl of Primary P3 solution buffer (Lonza) pre-mixed with RNP complex and enhancer for a final volume of 20 μl. Resuspended cells were electroporated in a 16-well nucleocuvette strip or 384 HT nucleocuvette system using program CM-138. Immediately after electroporation, 80 μl culture media was added on top of the cells before transferring to pre-warmed cell culture flasks.

### Genotyping analysis by Sanger sequencing

PCR of the genomic region flanking the CRISPR target site (~ 500 bp) and Sanger sequencing was carried out to assess editing efficiencies at the cell pool level. Three days after electroporation, cells were collected and resuspended in lysis buffer (DirectPCR lysis buffer (Viagen Biotech); 0.4 mg/ml proteinase K (Qiagen)) at a ratio of 10,000 cells per 10 μl lysis buffer. Cells were incubated for 2 h at 55 °C and 30 min at 85 °C to generate cell lysates. 2 μl of cell lysate was used in a 20 μl PCR reaction with 2 × Flash Phusion PCR Master Mix (Thermo Fisher) and 0.5 μM of custom forward and reverse primers (Sigma). The PCR products were amplified using 63 °C annealing temperature. Amplified products were sequenced in Eurofins and sequencing reads were analysed using both TIDE analysis (internal AZ v3 adapted from version 1.1.2 created by Bas van Steensel lab) and online ICE analysis tool (Synthego; https://ice.synthego.com/).

### Statistics

For in vivo studies**,** data were expressed as means ± SEM, and assessed for significance by Student's *t* test or ANOVA as appropriate. Patient demographics were compared using Student’s *t* test or Mann Whitney analysis. Categorical variables were compared using Fisher’s exact test. P values were determined for multiple comparisons using the Bonferroni correction. Values of *P ≤ 0.05, **P ≤ 0.01, ***P ≤ 0.005, ****P ≤ 0.001 were considered significant.

## Results

### Enhanced UPR in IPF lung samples and primary lung fibroblasts

**Figure 1:**
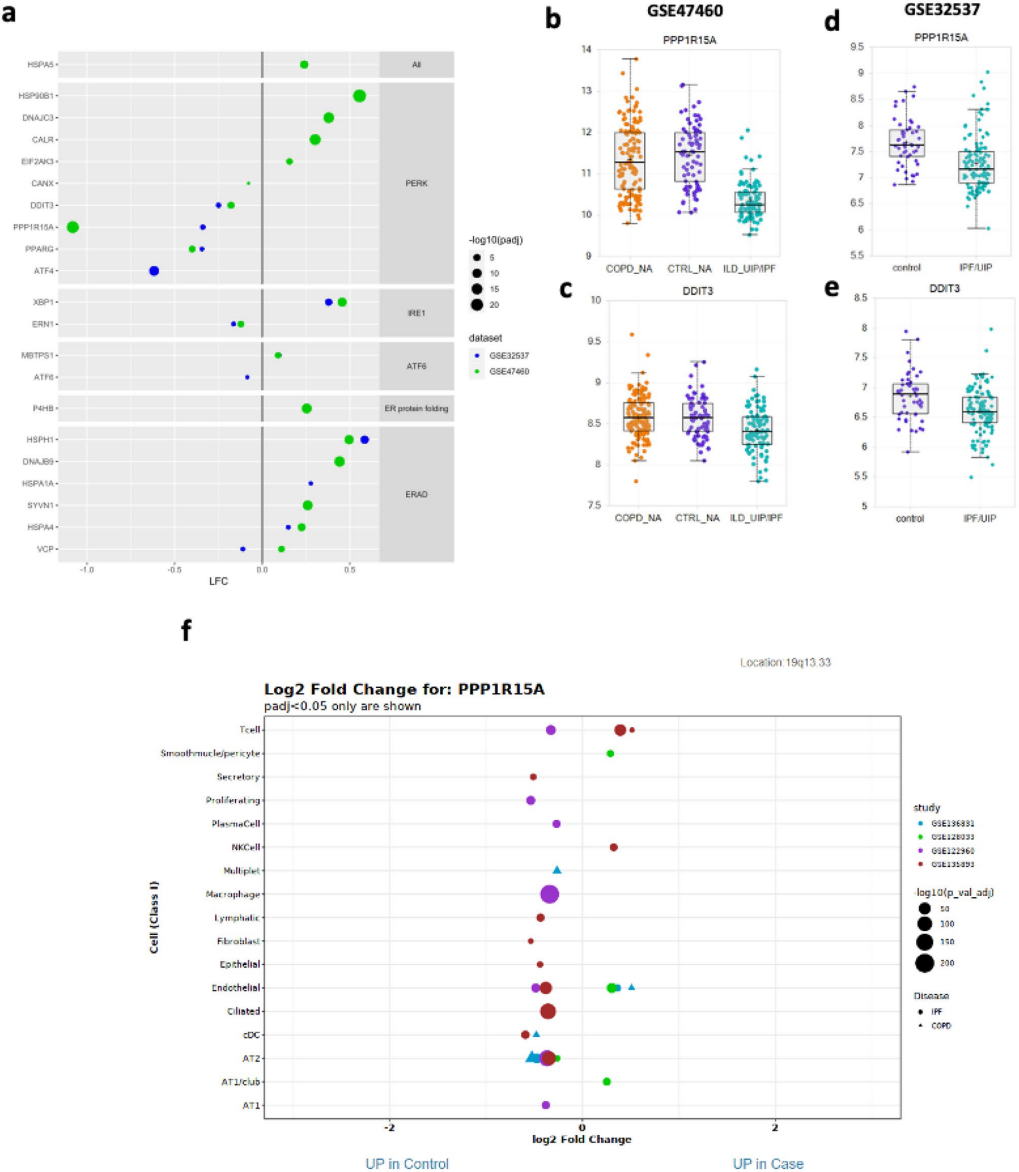
Altered expression of UPR genes in IPF lung tissues. (**a**) Plot illustrating IPF versus healthy control differential expression for 2 large cohort studies (GSE32537 (blue) and GSE47460 (green) for UPR genes; x-axis is log2 fold change (LFC), the size of the bubble indicates the − log10 FDR adjusted P-value (padj). The genes shown are grouped by pathway (shown to right, also in Table S1). While the majority of UPR genes are up-regulated in IPF lung compared to control, most of the genes in the ATF4 pathway, including PPP1R15A, are down-regulated in IPF. Genes shown but with no bubble displayed have data present but are not significant at FDR adjusted P-values < 0.1. (**b**–**e**) Boxplots of PPP1R15A and DDIT3 for the 2 cohort studies, where GSE47460 shows the expression of control and IPF/UIP samples (**b**,**c**) while GSE32537 also includes samples from COPD as well as control and IPF lung tissue (**d**,**e**). (**f**) Plot showing the differential expression of PPP1R15A across multiple cell types based on single cell RNA sequencing data. Cell types are listed on the y-axis and the log2 fold change for PPP1R15A for each cell type from each study (individual bubble) is on the x-axis. Legend for bubble colour and size is on the righ of the plot. Only samples with adjusted P-values < 0.05 are shown on the plot.

To confirm the presence of UPR in IPF, we profiled the expression of genes from the PERK, *IRE1* and *ATF6* branches of the UPR pathway (Fig. S1, Table S1) in two large cohort IPF genomic studies (Fig. 1a). This analysis highlighted that most genes in this pathway were differentially expressed in IPF as compared to healthy controls or individuals with chronic obstructive airways disease (COPD). For example, many genes were up-regulated, including the unfolded protein sensor BiP (*HSPA5*), PERK (*EIF2AK3*, the mediator of the translation arm of the UPR) along with many of the components of the endoplasmic-reticulum-associated protein degradation (ERAD) complex that lies downstream of both the IRE1/ERN1 and ATF6 UPR pathways. Of those genes down-regulated in IPF, the majority were those transcriptionally regulated by ATF4 and include *PPP1R15A*, *DDIT3*, and *ATF4* itself. *PPP1R15A* was the UPR gene with the largest decrease in IPF (i.e. over twofold in GSE47460 as well as being decreased in GSE32537) (Fig. 1b,d). *DDIT3*, an upstream regulator of PPP1R15A and downstream of ATF4 was also reduced in IPF in both GSE47460 (Fig. 1c) and GSE32537 (Fig. 1e).

The recent publication of multiple lung single cell IPF studies^25–28^ has allowed us to further determine which cell types may contribute to the IPF gene expression changes described above. We reprocessed the counts data from all four IPF scRNAseq studies and generated differential expression data for each of the cell type defined by the authors of each study (Fig. 1f and Table S2). PPP1R15A was found to be decreased in IPF fibroblasts as well as many cell types (Fig. 1f). DDIT3 was not found to be significantly altered in fibroblast cells but was down in IPF epithelial cell types, while ATF4 was also down in IPF epithelial cells and other immune cells but up in IPF fibroblasts (Table S2).

**Figure 2:**
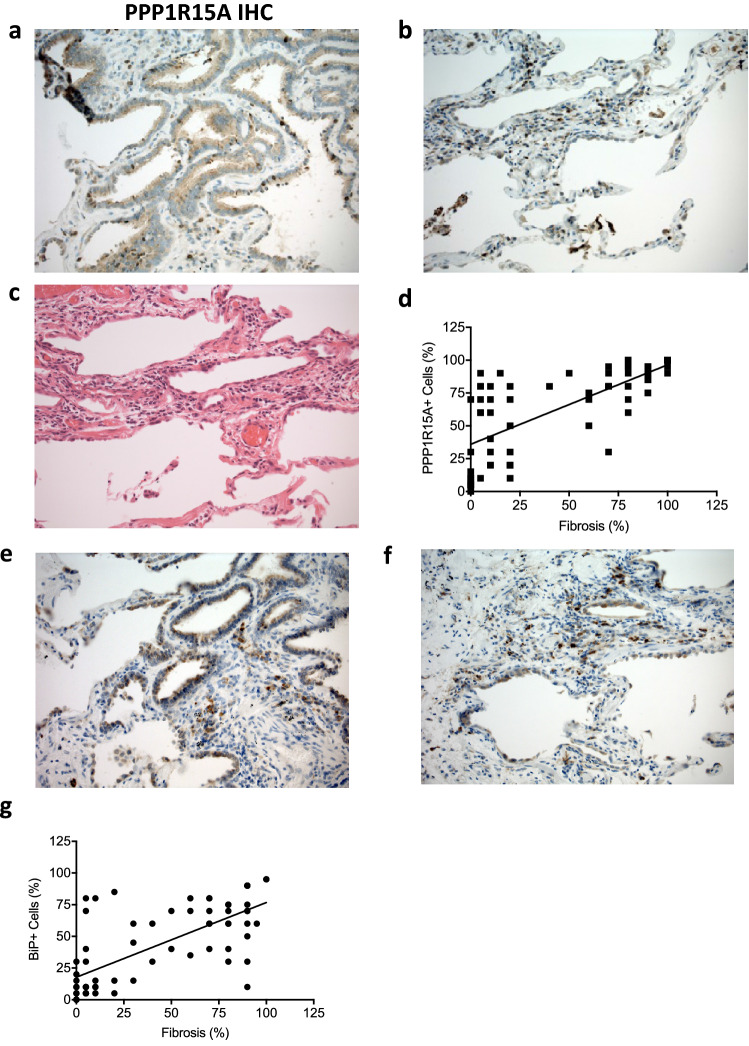
Immunolocalisation of ER stress markers. PPP1R15A (**a**,**b**) and BiP (**e**,**f**) in IPF human lung tissue and correlation of number of positive cells with extent of fibrosis. Representative images obtained using light microscopy (× 200 magnification) stained with rabbit polyclonal anti-PPP1R15A (**a**,**b**), haematoxylin and eosin (**c**), anti-BiP (**e**,**f**) shown. Correlation of PPPIR15A (**d**, r^2^ = 0.289, P < 0.001) and BiP (**g**, r^2^ = 0.408, P < 0.001) with extent of fibrosis in 4 UIP patients using semi-quantitative analysis in six high powered fields randomly selected by 2 blinded independent investigators. Data were analysed by linear regression.

Assessment of protein level expression of PPP1R15A and BiP by immunohistochemistry of IPF lung sections identified immunoreactive staining for both PPPIR15A (Fig. 2a-c) and BiP (Fig. 2e,f) within areas of fibrosis localised to reactive type II pneumocytes and columnar epithelium. There was minimal staining of either protein in the endothelium and alveolar macrophages. No PPPIR15A was evident in the mesenchymal cells (Fig. 2b). There were significant associations with epithelial PPP1R15A expression and fibrosis (Fig. 2d, r^2^ = 0.289, P < 0.001) and epithelial BiP to fibrosis (Fig. 2g, r^2^ = 0.408, P < 0.001). There was no correlation with PPP1R15A or BiP with areas of inflammation (data not shown).

**Figure 3:**
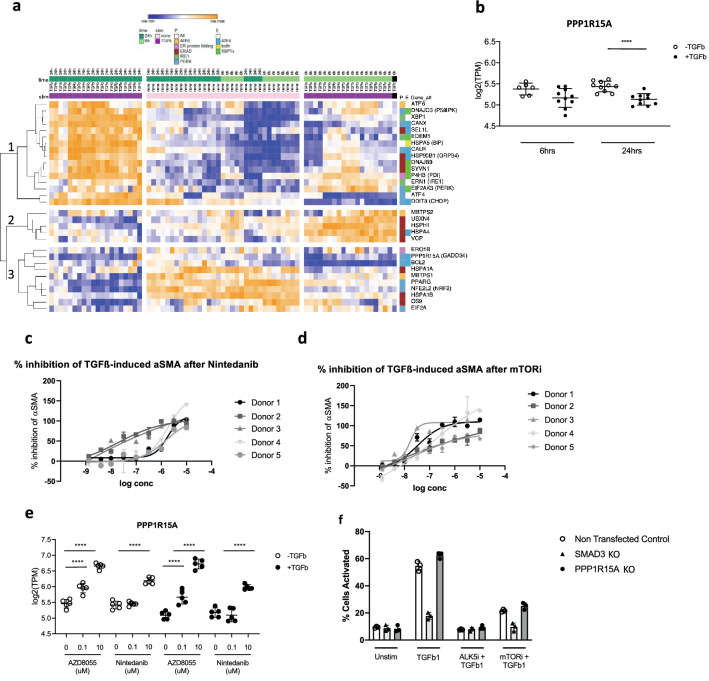
Modulation of TGFβ1-mediated UPR pathway and fibroblast activation. (**a**) Heatmap of gene expression [log2(TPM)] of the UPR genes from Table S1 for IPF fibroblasts (5 donors, 3–5 replicates) treated with TGFβ1 (0.123 ng/ml) for 6 h or 24 h. The genes are further colour coded into pathway (P) and expression regulators (E) according to Table S1. The genes fall into three groups based on their expression: those genes whose expression is repressed by TGFβ1 at either or both times including PPP1R15A (bottom panel); those genes whose expression is only transiently up-regulated by TGFβ at 6 h (middle panel); and those genes including DDIT3 and ATF4 whose expression is up-regulated by TGFβ at 24 h (top panel). This last group of genes consist of all genes regulated by XBP1 and the majority of those regulated by ATF4. (**b**) Expression plots of PPP1R15A (**b**) following TGFβ1 treatment at both 6 h and 24 h. (**c**,**d**) Dose–response curves for IPF fibroblasts treated with TGFβ1 (0.123 ng/ml) in the presence or absence of different concentrations of nintedanib (**c**) or mTOR inhibitor (AZD8055) (**d**) for 24 h. The expression of α-SMA was determined by immunofluorescence. (**e**) Gene expression of PPP1R15A in IPF fibroblasts in the presence or absence of TGFβ1 plus nintedanib or AZD8055 for 24 h. n = 5 IPF donor cell lines with each dot representing a different donor in each condition and data is expressed as mean ± s.e.m. (**f**) TGFβ-induced myofibroblast activation was quantified between KOs and wild-type (non-transfected control: NTC) cells. Cells in starvation conditions were incubated with positive control compounds (mTOR inhibitor and ALK5 inhibitor) (3 µM) for 1 h before treatment with TGFβ (0.125 ng/ml) for 24 h. Unstimulated cells were treated with 0.1% (v/v) DMSO for normalisation. SMAD3 KO was used as a positive control for inhibition of TGFβ-induced myofibroblast activation. Myofibroblast activation was measured by immunofluorescence of αSMA and represented as percent cell activation, based on Columbus linear classifier algorithms using intensity, texture and morphology features. FDR adjusted P-values are show on plots where significant, **P < 0.01, ****P < 0.0001.

Because of the major pathologic role of fibroblasts in IPF, we exposed primary IPF fibroblasts to TGFβ1 (n = 5 donors) and determined transcriptomic changes in UPR gene expression at both 6 h and 24 h after addition of cytokine (Fig. 3a). We observed that the UPR-related transcripts could be organized into 3 main groups: (1) those that show a transient, early (6 h) increase in response to TGFβ1 and comprises genes mainly in the ERAD complex (Fig. 3a, group 2); (2) transcripts induced by TGFβ1 at 24 h (and in some cases also at 6 h) which included *DDIT3*, *ATF4* and other genes whose expression was regulated by either of these (Fig. 3a, group 1); and (3) genes whose expression was decreased by TGFβ1 at 24 h (and in some cases at 6 h also) which includes *PPP1R15A* (Fig. 3b), as well as *BCL2* and *NRF2* (group 3, Fig. 3a). TGFβ inhibited *PPP1R15A* expression, with a trend observed at 6 h and significant inhibition observed at 24 h (Fig. 3b). PPP1R15A protein was measured by western blot in human lung fibroblasts stimulated with TGFβ1 in order to confirm that changes in gene expression also can be detected at the protein level. PPP1R15A protein is undetectable by western blot in the fibroblast cell lysates (Fig. S2), and there is a clear increase upon stimulation with the ER stress inducer tunicamycin. In contrast, TGFβ1 stimulation does not induce an increase in PPP1R15A, and is undetectable in these cells (Fig. S2). Additional protein analyses indicated that CHOP was undetectable in unstimulated cells or TGFβ stimulated cells, but upregulated by tunicamycin (Fig. S2). BiP was detectable in unstimulated cells and increased by tunicamycin, but not modulated by TGFβ (Fig. S2). Furthermore, p-eIF2a was only slightly increased by TGFβ (Fig. S2).

### Both nintedanib and AZD8055 inhibit the effect of TGFβ1 on IPF fibroblasts

TGFβ1 significantly promoted fibroblast to myofibroblast transition in IPF cells at 24 h, as shown by increased α-smooth muscle actin (αSMA) (Fig. 3c), and pre-treatment with nintedanib attenuated this effect in a dose- and donor-dependent manner. Nintedanib has previously been shown to inhibit TGFβ responses^29^. Additionally, as previously reported^30^, preincubation with the mTOR inhibitor AZD8055 also attenuated the TGFβ1 response (Fig. 3d). However again, this modulation was donor dependent and differed between donors.

Transcriptomic analysis of these same cells indicated that both nintedanib and AZD8055 were able to suppress many of the genes downstream of PERK/eIF2α and IRE1/ERN1 induced by TGFβ1 in primary lung fibroblasts (Fig. S3). Nintedanib (at 10 µM) enhanced the expression of *PPP1R15A* and reversed TGFβ1-mediated suppression of this factor (Fig. 3e). In contrast, AZD8055 (at 0.1 µM) promoted the expression of *PPP1R15A* to baseline levels and further induced gene expression at 10 µM (Fig. 3e). We next assessed the role of PPPIR15A in TGFβ-mediated responses in fibroblasts using CRISPR-based gene knockdown in the same assay. In these experiments, we observed that CRISPR-mediated *PPPIR15A* knockdown had no impact on TGFβ-induced fibroblast activation, nor did it modulate mTOR-mediated inhibition of TGFβ-induced *ACTA2* induction (Fig. 3f, Fig. S4). Together, these data suggested that both nintedanib and AZD8055 enhanced *PPP1R15A* expression but the effects of these drugs on TGF-β-induced fibroblast activation was likely due to *PPP1R15A*-independent mechanisms.

### Bleomycin-induced lung fibrosis correlates with senescence and ER stress

**Figure 4:**
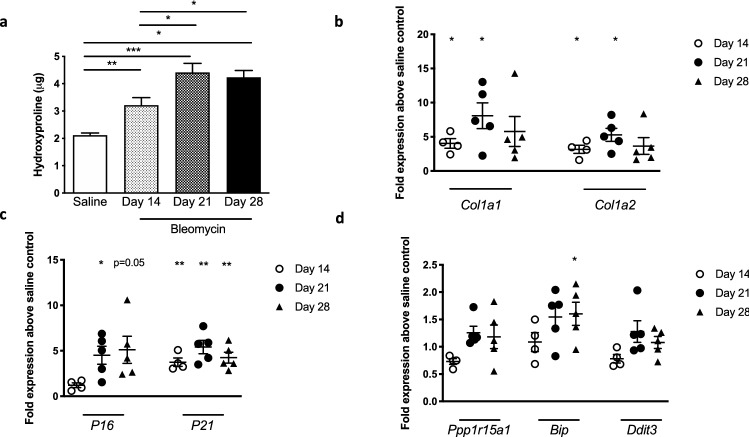
UPR and cellular senescence markers correlate with fibrosis in experimental bleomycin-induced pulmonary fibrosis. (**a**) Hydroxyproline levels in the lungs of C57Bl/6 mice at designated timepoints after oropharyngeal bleomycin challenge (n = 6–8/ group). (**b**–**d**) Gene expression as measured by RT-PCR of fibrosis-related genes (**b**), senescence associated markers (**c**) and UPR-pathway related genes (**d**) in whole lung tissue following bleomycin challenge as measured as fold change in gene expression in the bleomycin challenged mice in comparison to saline-challenged control mice (n = 4–5 per group). Data are shown as mean ± s.e.m. *P < 0.05, **P < 0.01, ***P < 0.001 as designated or, when no bars, as compared to saline control mice.

Intrapulmonary bleomycin delivery to mice results in classic acute inflammatory response followed by increased fibrosis in the lung. In this experiment, the fibrotic response as measured by whole lung hydroxyproline levels (Fig. 4a) and matrix-associated gene expression (Fig. 4b) was present at days 14, 21 and 28 after bleomycin administration. In addition, we observed an increase in cellular senescence markers including *P16* and *P21* gene expression at these times after bleomycin challenge (Fig. 4c). Interestingly, we also observed alterations in *Ppp1r15a*, *Bip* and *Ddit3* gene expression over time following bleomycin administration (Fig. 4d) in the lungs of these mice, with *Ppp1r15a* and *Ddit3* showing differences in expression when comparing day 14 and day 21 after bleomycin.

### Genetic deletion of *Ppp1r15a* exacerbates bleomycin-induced lung fibrosis and inflammation

**Figure 5:**
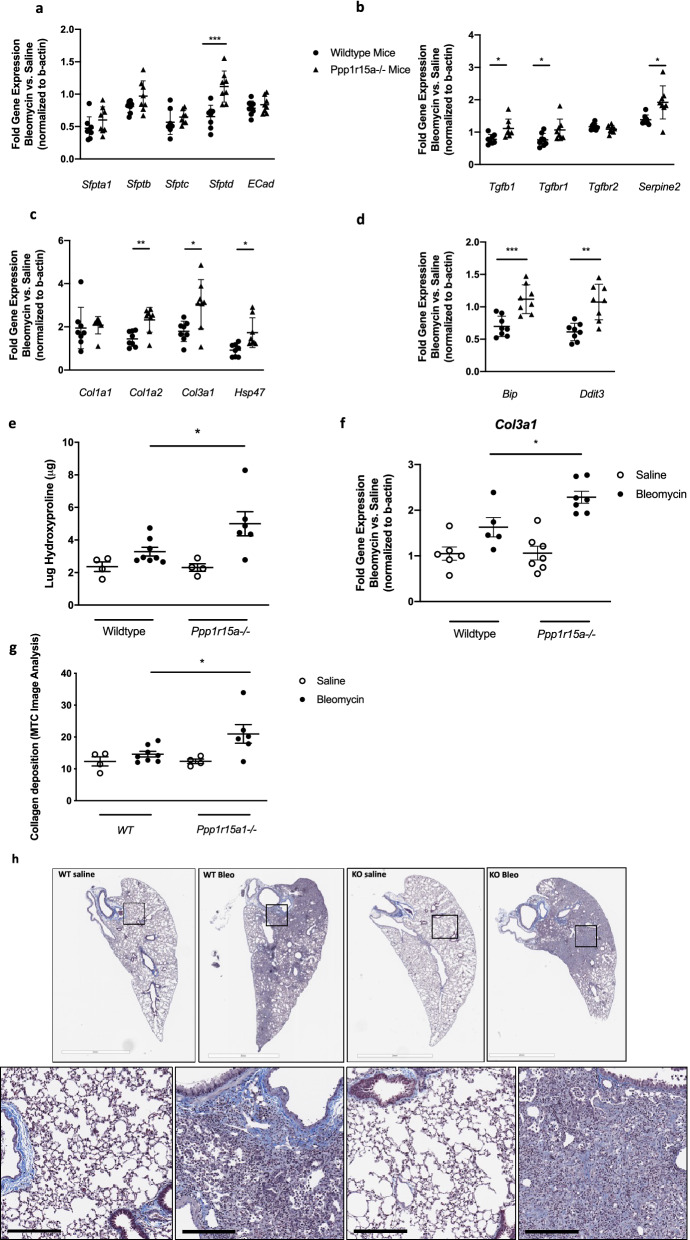
Loss of PPP1R15A exacerbates bleomycin-induced lung fibrosis. (**a**–**d**) Whole lung gene expression of epithelial genes (**a**), TGFβ- and senescence-related genes (**b**), collagen-related genes (**c**), and UPR/ER stress related genes (**d**) following oropharyngeal bleomycin administration to *Ppp1r15a*^−/−^ and wildtype littermate controls at Day 5 after bleomycin administration. (**e**–**h**) Hydroxyproline levels in the lungs of *Ppp1r15a*^−/−^ and wildtype littermate controls challenged with oropharyngeal bleomycin or saline (n = 4–7/group) measured at Day 21 and quantified by biochemical analysis (**e**), whole lung *Col3a1* gene expression as measured by RT-PCR (**f**), and histological quantification of Masson’s Trichrome stained histological sections (**g**), with representative images shown in (**h**). Data shown are mean ± s.e.m. fold change in gene expression mediated by bleomycin in comparison to relevant littermate control. Data are shown as mean ± s.e.m. *P < 0.05, **P < 0.01, ***P < 0.001 as designated or when no bars, as compared to saline control mice.

To gain mechanistic insights into the exacerbated fibrotic response observed in absence of *Ppp1r15a*, we next profiled the acute response to bleomycin in *Ppp1r15a*^−/−^ mice and wildtype (wt) littermate controls at day 5 after intrapulmonary bleomycin. In assessing the expression of epithelial-related genes including the surfactant proteins and e-cadherin after bleomycin, we observed significantly increased surfactant protein d (*Sfptd*) but comparable modulation of other epithelial-related genes assessed in both wt and *Ppp1r15a*^−/−^ mice (Fig. 5a). *Ppp1r15a*^−/−^ mice also had increased *Tgfb1*, *Tgfbr1* and the senescence-associated gene *Serpine2* in response to bleomycin in comparison to wt controls (Fig. 5b). We also observed an increase in the bleomycin-induced fibrosis-related genes including *Col1a2*, *Col3a1* and the collagen chaperone *Hsp47* (Fig. 5c). There was no detectable *P16* or *P21* in the lung at day 5 after bleomycin (data not shown), hence we focused on *Serpine2* expression in our assessment of changes in cellular senescence. Interestingly, bleomycin challenged *Ppp1r15a*^−/−^ mice exhibited altered expression in a number of ER stress/UPR-related genes at day 5 compared to similarly bleomycin challenged wt mice (Fig. 5d). We confirmed that the augmented response to bleomycin was not due to increased bleomycin delivery to the *Ppp1r15a*^−/−^ mice as both groups of mice had a comparable induction of *Mmp12* in the lungs at both days 5 and 21 after bleomycin (Fig. S5a). There was however an increase in lung TNFa (Fig. S6a) and a trend towards an increase in lung IL1B (Figs. S5, S6) in bleomycin challenged *Ppp1r15a*^−/−^ mice.

To determine whether the exacerbated responses observed in the *Ppp1r15a*^−/−^ mice in the response to bleomycin impacted fibrosis, we next challenged *Ppp1r15a*^−/−^ mice with low dose bleomycin since we hypothesized that the *Ppp1r15a*^−/−^ mice would be more sensitive to bleomycin challenge. We initially compared their responses to those of bleomycin challenged wt mice at day 21. There was no detectable *Ppp1r15a* in the lungs of *Ppp1r15a-/-* at any time point assessed (data not shown). As hypothesized, *Ppp1r15a*^−/−^ mice had significantly enhanced lung hydroxyproline content (Fig. 5e) and *Col3a1* gene expression (Fig. 5f). Furthermore, image analysis of Masson’s Trichrome-stained lung sections further revealed an exacerbated response to bleomycin (Fig. 5g,h). There was also an increase in whole lung tissue TNFα protein levels, as measured by MSD analysis (Fig. S5b).

**Figure 6:**
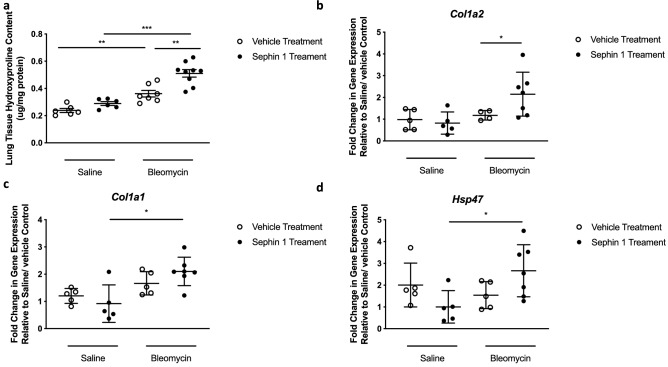
Pharmacological inhibition of PPP1R15A exacerbates bleomycin-induced lung fibrosis. C57Bl6 mice received oropharyngeal bleomycin or saline on Day 0 and were treated daily with Sephin1 (5 mg/kg, p.o.) or vehicle control (saline, 10 ml/kg p.o.) on Days 14–20. Mice were killed on Day 21 and lungs analysed for total collagen content via hydroxyproline assay (**a**) and total lung gene expression quantified for *Col1a1* (**b**), *Col1a2* (**c**) and *Hsp47* (**d**) via RT-PCR. Data are shown as mean ± s.e.m. *P < 0.05, **P < 0.01, ***P < 0.001 as designated.

To further confirm the role of PPP1R15A in the bleomycin model, we targeted PPP1R15A with the small molecule inhibitor, Sephin1^31^. C57Bl/6 mice (i.e. wt) were administered a low dose of bleomycin and therapeutically dosed with Sephin1 daily from days 14 to 20 after bleomycin, thus avoiding the acute inflammatory features of the model. Like bleomycin challenged *Ppp1r15a*^−/−^ mice, wt mice treated with Sephin1 had significantly increased lung fibrosis at day 21 compared with the vehicle control treated mouse group, as measured by lung tissue hydroxyproline (Fig. 6a), and whole lung gene expression of *Col1a2* (Fig. 6b). Interestingly it was only the Sephin1-treated bleomycin-challenged mice that exhibited elevated *Col1a1* (Fig. 6c) and *Hsp47* (Fig. 6d) compared to Sephin1-treated control mice, with bleomycin not eliciting a significant increase in *Col1a1* or *Hsp47* in vehicle-treated animals.

### Loss of PPP1R15A accelerates fibroblast senescence

**Figure 7:**
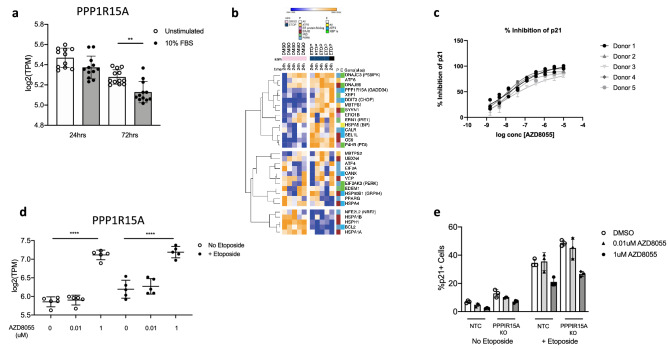
Etoposide-induced senescence in IPF lung fibroblasts is inhibited by the mTOR inhibitor AZD8055. (**a**) Expression of PPP1R15A in NHLF 24 h and 74 h after stimulation with 10% FBS. (**b**) Heatmap of gene expression [log2(TPM)] of the UPR genes from Table S1 for IPF fibroblasts (5 donors) treated with etoposide (3 µM) for 24 h. The genes are further colour coded into pathway (P) and expression regulators (E) according to Table S1. Top panel are genes induced by etoposide including PPP1R15A, bottom panel are genes suppressed by etoposide. (**c**) Dose–response curves for AZD8055. IPF fibroblasts were cultured in the presence of etoposide (3 µM) and with the indicated compound concentrations for 72 h, after which the cells were stained with an anti-p21 antibody as well as the cell and nucleus stains CellMask and Hoechst. The cells were imaged and the p21 inhibition percentage with AZD8055 was determined using image analysis. (**d**) Gene expression of PPP1R15A in IPF fibroblasts in the presence or absence of etoposide plus AZD8055 for 24 h. n = 5 IPF donor cell lines with each dot representing a different donor in each condition. (**e**) Etoposide-induced senescence was quantified between PPP1R15A KOs and wild-type (non-transfected control: NTC) cells. Cells were treated with AZD8055 (0.01 μM or 1 μM) and etoposide (3 μM) for 72 h. Senescence quantification was measured by immunofluorescence of nuclear p21 and represented as percentage p21 positive cells. Data is expressed as mean ± s.e.m. FDR adjusted P-values are show on plots where significant, *P < 0.05, ****P < 0.0001.

*PPP1R15A* was reduced in proliferating, normal human lung fibroblasts maintained in 10% FBS for 24 or 72 h (Fig. 7a). Treatment of IPF fibroblasts with etoposide increased many UPR genes including both *PPP1R15A* and *XBP1* (top section of heatmap Fig. 7b). A smaller group of mostly ERAD genes were repressed by etoposide (bottom part of Fig. 7b). The remaining genes show variable responses. We next assessed the effects of nintedanib and AZD8055 on IPF fibroblast senescence. Nintedanib had no effect on etoposide-induced cellular senescence (Fig. S7). However, inhibition of mTOR with AZD8055 has been previously reported to impact cellular senescence^32^, and in this study AZD8055 attenuated the effect of etoposide on p21 expression (Fig. 7c) and on the other senescence endpoints of cell and nuclear area (Fig. S8). Interestingly, unlike the TGFβ1 assay, all IPF donor cell lines responded similarly to AZD8055. Etoposide upregulated *PPP1R15A*, however AZD8055 induced *PPP1R15A* in both unstimulated and etoposide-stimulated cells (Fig. 7d). CRISPR-mediated PPP1R15A knockdown induced cellular senescence directly, as observed by increased p21^+^ fibroblast number, and further promoted etoposide-induced cellular senescence (Fig. 7e). However, mTOR inhibition mediated reduction of senescence was independent of *PPPIR15A* (Fig. 7e).

## Discussion

Herein, we demonstrate that IPF is associated with altered expression of the UPR pathway in lung mesenchymal cells. We show that the UPR/ER stress pathway is altered in two large transcriptomics studies employing IPF lung samples^22,23^ and in both studies the *PERK*/*EIF2AK3* pathway and the *IRE1* pathway were activated in IPF compared with normal lung (Fig. 1). The key roles for these pathways in controlling the UPR are to maintain tissue homeostasis via attenuating protein translation and facilitating cytoprotection^33^. Recently, the ATF4 pathways has also been implicated in mitochondrial dysfunction, which then drives ER stress and subsequent lung pathology in preclinical lung damage models^34^. The loss of PPP1R15A promotes continual phosphorylation of eIF2a, turning off protein synthesis, which in IPF could prevent normal alveolar repair. Through a detailed gene-based analysis of whole lung and single cell RNAseq, we demonstrate that a key component of the UPR pathway PPP1R15A is reduced in IPF fibroblasts compared with normal lung fibroblasts. Furthermore, using both genetic deletion or pharmacologically based inhibition of PPP1R15A, we demonstrate that reduced PPP1R15A activity exacerbated bleomycin-induced lung fibrosis. Mechanistically, we linked ER stress to senescence in lung fibroblasts. Specifically, PPP1R15A is involved in cellular senescence since it’s deletion in fibroblasts resulted in increased p21 and exacerbated etoposide-induced senescence of these mesenchymal cells. Together, these data demonstrate that reduced PPP1R15A enhances lung fibroblast to myofibroblast differentiation and accelerates the senescence of these cells in lung fibrosis. Modulation of PPP1R15A might provide therapeutic benefit in IPF via direct effects on lung fibroblasts.

It has been hypothesized that IPF is a consequence of chronic and unabated epithelial injury which then maintains the aberrant wound healing response of excess fibroblast-mediated matrix deposition. Consequently, most studies linking ER stress and IPF have predominantly focused on the epithelium^8^. One such link is via surfactant proteins, which are processed via the endoplasmic reticulum^35,36^. Familial IPF has been linked with mutations in the surfactant protein genes, SFTPC^37^ and SFTPA2^38^. These mutations are thought to result in ER stress and UPR pathway activation in AT2 cells^38–41^. Other potential inducers of ER stress that are relevant to IPF include viral infection including herpesvirus such as Epstein Barr Virus (EBV) or cytomegalovirus (CMV), which are commonly detected in IPF lung and not healthy lung tissue^8,41^, and have been linked to fibrosis development^42^. Additional key drivers of ER stress in IPF include oxidative stress and environmental pollution^2,43^, and oxidative stress mediated ER stress has been linked to fibroblast differentiation induced by TGFβ^9^.

Our studies point to a distinct role for PPP1R15A in lung fibroblasts that is apparent in both the proliferative and senescent state of these cells. In a proliferative state, lung fibroblasts exhibit a relative loss of PPP1R15A as revealed in immunohistochemical analysis of advanced fibrotic areas such as fibroblastic foci. Moreover, the relative loss was greater when whole lung samples were analysed in the large cohort transcriptomic studies. The absence of PPP1R15A in lung fibroblasts enhanced the response of these cells to TGF-β and enhanced expression of PPP1R15A was associated with diminished response to this pro-fibrotic cytokine. Another downstream response following oxidative stress is cellular senescence. Elevated epithelial and fibroblast senescence has been observed in the lungs of IPF patients^15,16^ and this process has been more broadly linked to premature ageing^44^ and promoting lung fibrosis^45,46^. Importantly, targeting cellular senescence has resulted in therapeutic benefit in preclinical models of lung fibrosis^47,48^. Etoposide is a commonly used agent for mediating fibroblast senescence, by eliciting DNA damage^49^. Previously we have reported an impairment in the DNA damage response in IPF fibroblasts^16^. In the present study, we used etoposide as an in vitro model to assess the role of PPP1R15A. A recent paper has highlighted the significance of the mTOR pathway in regulating fibrosis via senescence, as well as inhibiting TGFβ-induced collagen production^30^. Here we observed that mTOR pathway inhibition with AZD8055 inhibited etoposide-induced fibroblast senescence. PPP1R15A knockdown resulted in an increase in fibroblast senescence in resting fibroblasts and a further increase in etoposide-induced p21 expression. However, the mTOR-mediated effect on senescence pathway is independent of PPP1R15A.

The potential dual role of PPP1R15A is also apparent depending on the extent of injury to the lung. In settings of mild injury such as following acrolein challenge, PPP deficiency will enable misfolded proteins to be cleared and a coordinated repair response to occur^50^. However, if the injury is overwhelming, such as demonstrated in our study following bleomycin administration, PPP1R15A deficiency resulted in exacerbated lung damage and fibrosis. Notwithstanding the bleomycin model spontaneously resolves in healthy, young mice^16^, in both the gene-deficient and Sephin1 treated mice challenged with bleomycin this was augmented in lung fibrosis. The mechanism through which PPP1R15A deficient mice exhibit exacerbated fibrosis might be due to a reduction in this resolution process as both col1a2 and hsp47, a collagen chaperone, are involved in stabilizing mature collagen^51^. Another mechanism by which PPP1R15A deficiency might be promoting overall lung fibrosis is via TGFβ receptor phosphorylation. A previous report indicated that PPP1R15A dephosphorylates TGFβ type 1 receptor^52^. Herein, we observed an increase in bleomycin induced TGFβ1 and the type I receptor at day 5 after bleomycin in the Ppp1r15a gene deficient animals, however we did not observe an exacerbation of TGFβ induced effects in the fibroblast CRISPR model suggesting that any TGFβ receptor-associated mechanisms are fibroblast-independent in lung fibrosis. Thus, future studies looking at extended timepoints and also confirming specific genes that are modulated at the protein level, this may indicate that although the acute inflammation and subsequent collagen deposition is exacerbated, the resolution processes may also be accelerated.

Overall, our study highlights a potential protective role for PPP1R15A in both the initiation and maintenance of lung fibrosis. The protective role appears to encompass both proliferating and senescent lung fibroblasts. In light of our novel findings, we speculate that the restoration of PPP1R15A activity in lung fibroblasts might represent an attractive therapeutic approach that interrupts the relentless deposition of extracellular matrix in IPF.

## Supplementary Information

Supplementary Tables.

Supplementary Information.
