# Supplementary material for: Sensitization of the UPR by loss of PPP1R15A promotes fibrosis and senescence in IPF
Source: Sci Rep. 2021 Nov 3;11:21584. doi: 10.1038/s41598-021-00769-7 (PMC8566588; doi:10.1038/s41598-021-00769-7)
Supplement: Supplementary file 1 — Supplementary Tables. [file 41598_2021_769_MOESM1_ESM.pdf]

Pathway	Symbol	alias	Gene Name	Features	Expression regulated by (IPA#)	activity regulated by
**All**	HSPA5	BIP/ GRP78	heat shock protein family A (Hsp70) member 5	unfolded protein sensor	ATF4, ABP1, and ATF6	
**PERK**	EIF2AK3	PERK	eukaryotic translation initiation factor 2 alpha kinase 3	inhibited by BIP	XBP1s	
**PERK**	DNAJC3	P58IPK	DnaJ heat shock protein family (Hsp40) member C3	inhibitor of PERK	XBP1s	
**PERK**	NFE2L2	NRF2	nuclear factor, erythroid 2 like 2			
**PERK**	EIF2A		eukaryotic translation initiation factor 2A	regulator of translation, activated by phos by PERK, inhibited by dephos by PPP1R15A		
**PERK**	ATF4		activating transcription factor 4	downstream of EIF2a		
**PERK**	BCL2		BCL2 apoptosis regulator	target of CHOP	ATF4	
**PERK**	CALR		calreticulin	downstream of ATF4	ATF4	
**PERK**	CANX		calnexin	downstream of ATF4	ATF4	
**PERK**	DDIT3	CHOP	DNA damage inducible transcript 3	downstream of ATF4 mediator of apoptotic response1	ATF4	
**PERK**	HSP90B1	GRP94	heat shock protein 90 beta family member 1	downstream of ATF4- ERAD component	ATF4	
**PERK**	PPP1R15A	GADD34	protein phosphatase 1 regulatory subunit 15A	inhibits EIF2a	ATF4, DDIT3	
**PERK**	PPARG	PPARγ	peroxisome proliferator activated receptor gamma	downstream EIF2a		
**ATF6**	ATF6		activating transcription factor 6	in ER, transcription factor		
**ATF6**	MBTPS1		membrane bound transcription factor peptidase, site 1	golgi TM protein cleaves ATF6 to nuclear form		
**ATF6**	MBTPS2		membrane bound transcription factor peptidase, site 2	golgi TM protein cleaves ATF6 to nuclear form		
**IRE1**	ERN1	IRE1	endoplasmic reticulum to nucleus signaling 1	ER TM kinase and splicing factor, activates XBP1		
**IRE1**	XBP1		X-box binding protein 1	TF downstream of IRE1, interacts w CEBPs		ERN1/IRE1
**IRE1**	EDEM1		ER degradation enhancing alpha-mannosidase like protein 1	downstream of XBP1	XBP1s	XBP1
**ERAD**	DNAJB9		DnaJ heat shock protein family (Hsp40) member B9	HSP40 member- (co-chaperone for Hsp70 protein HSPA5/BiP that acts as a key repressor of the ERN1/IRE1-mediated unfolded protein response)	XBP1	
**ERAD**	HSPA1A		heat shock protein family A (Hsp70) member 1A	HSP70 member co-chaperone of BIP downstream of XBP1(ATF6)-ERAD		
**ERAD**	HSPA1B		heat shock protein family A (Hsp70) member 1B	HSP70 member co-chaperone of BIP downstream of XBP1(ATF6)-ERAD		
**ERAD**	HSPA4	HSP70	heat shock protein family A (Hsp70) member 4	HSP70 member co-chaperone of BIP downstream of XBP1(ATF6)-ERAD	ATF4	
**ERAD**	HSPH1		heat shock protein family H (Hsp110) member 1	part of degredation downstream of XBP1(ATF6)		
**ERAD**	OS9		OS9 endoplasmic reticulum lectin	ERAD component		
**ERAD**	SEL1L		SEL1L adaptor subunit of ERAD E3 ubiquitin ligase	ERAD component	ATF4	
**ERAD**	SYVN1		synoviolin 1	ERAD component	XBP1	
**ERAD**	UBXN4	Erasin	UBX domain protein 4	involved in protein degredation		
**ERAD**	VCP		valosin containing protein	involved in protein degredation, binds UBXN4		
**ER protein folding**	ERO1B		endoplasmic reticulum oxidoreductase 1 beta	activated by PDI- protein folding/unfolding		
**ER protein folding**	P4HB	PDI	prolyl 4-hydroxylase subunit beta	PDI component	XBP1s	

Table S1. Components of the unfolded protein response pathways. The genes of the various key components of the 3 main UPR pathways are listed along with their common aliases, and to which branch of the pathway they belong. Key features as well as what other pathway member regulates them (either transcriptionally or other means) are also listed. # Ingenuity Pathway Analysis (IPA; Qiagen)

Gene	avg_logFC	pct.1	pct.2	p_val_adj	pct.diff	dir	study	Cell(Author ID)	Class	Type
**ATF4**	0.27	0.37	0.16	4.77E-07	0.21	up	GSE128033	Fibroblast	Fibroblast	stromal
**ATF4**	0.43	0.16	0.10	2.29E-04	0.06	up	GSE128033	Smooth-mucle_or_pericyte	Smoothmucle/pericyte	stromal
**PPP1R15A**	-0.54	0.54	0.61	3.41E-02	-0.08	down	GSE135893	Fibroblasts	Fibroblast	stromal
**PPP1R15A**	0.29	0.39	0.19	4.83E-06	0.20	up	GSE128033	Smooth-mucle_or_pericyte	Smoothmucle/pericyte	stromal
**ATF4**	-0.72	0.28	0.69	2.02E-73	-0.41	down	GSE122960	CD8 T cells	Tcell	immune
**ATF4**	-0.68	0.59	0.83	2.40E-21	-0.24	down	GSE122960	Proliferating immune	Proliferating	immune
**ATF4**	-0.63	0.30	0.73	0.00E+00	-0.42	down	GSE122960	Alveolar.macrophages	Macrophage	immune
**ATF4**	-0.58	0.31	0.62	1.61E-10	-0.31	down	GSE122960	Mast cells	MastCell	immune
**ATF4**	-0.51	0.29	0.51	9.24E-02	-0.22	down	GSE122960	B cells	Bcell	immune
**ATF4**	-0.39	0.48	0.67	3.39E-09	-0.20	down	GSE122960	DC	DC	immune
**DDIT3**	-0.33	28%	42%	2.74E-02	-15%	down	GSE135893	Mast Cells	MastCell	immune
**DDIT3**	-0.26	18%	36%	9.73E-03	-18%	down	GSE135893	cDCs	cDC	immune
**PPP1R15A**	-0.59	0.65	0.83	2.28E-12	-0.18	down	GSE135893	cDCs	cDC	immune
**PPP1R15A**	-0.54	0.33	0.65	5.59E-17	-0.33	down	GSE122960	Proliferating immune	Proliferating	immune
**PPP1R15A**	-0.34	0.21	0.49	7.28E-201	-0.29	down	GSE122960	Alveolar.macrophages	Macrophage	immune
**PPP1R15A**	-0.33	0.13	0.41	1.07E-24	-0.28	down	GSE122960	CD8 T cells	Tcell	immune
**PPP1R15A**	-0.32	0.85	0.83	8.45E-02	0.02	up	GSE135893	Mast Cells	MastCell	immune
**PPP1R15A**	-0.27	0.20	0.41	1.54E-10	-0.21	down	GSE122960	Plasma cells	PlasmaCell	immune
**PPP1R15A**	0.32	0.59	0.48	2.49E-09	0.11	up	GSE135893	NK Cells	NKCell	immune
**PPP1R15A**	0.39	0.69	0.44	1.37E-47	0.25	up	GSE135893	T Cells	Tcell	immune
**PPP1R15A**	0.51	0.64	0.29	4.69E-02	0.36	up	GSE135893	Proliferating T Cells	Tcell	immune
**ATF4**	-0.70	0.36	0.75	0.00E+00	-0.38	down	GSE122960	AT2	AT2	epithelial
**ATF4**	-0.53	0.52	0.78	6.31E-19	-0.26	down	GSE122960	AT1	AT1	epithelial
**ATF4**	-0.35	0.39	0.57	9.28E-09	-0.18	down	GSE122960	Ciliated	Ciliated	epithelial
**ATF4**	-0.32	0.50	0.68	1.12E-04	-0.17	down	GSE122960	Secretory	Secretory	epithelial
**ATF4**	-0.27	0.35	0.52	5.07E-08	-0.17	down	GSE136831	ATII	AT2	epithelial
**ATF4**	0.27	0.28	0.10	1.90E-07	0.18	up	GSE128033	AT1_or_Club	AT1/club	epithelial
**DDIT3**	-1.1	14%	54%	2.40E-03	-39%	down	GSE135893	KRT5-KRT17+	Epithelial	epithelial
**DDIT3**	-0.36	23%	39%	4.04E-10	-16%	down	GSE135893	Transitional AT2	AT2	epithelial
**DDIT3**	-0.3	11%	31%	5.63E-08	-20%	down	GSE122960	AT1	AT1	epithelial
**DDIT3**	-0.27	6%	26%	8.53E-85	-20%	down	GSE122960	AT2	AT2	epithelial
**PPP1R15A**	-0.51	0.39	0.69	2.27E-04	-0.30	down	GSE135893	SCGB3A2+	Secretory	epithelial
**PPP1R15A**	-0.47	0.42	0.65	3.01E-24	-0.24	down	GSE136831	ATII	AT2	epithelial
**PPP1R15A**	-0.44	0.37	0.68	2.19E-03	-0.32	down	GSE135893	Proliferating Epithelial Cells	Epithelial	epithelial
**PPP1R15A**	-0.38	0.21	0.52	3.32E-11	-0.31	down	GSE122960	AT1	AT1	epithelial
**PPP1R15A**	-0.37	0.13	0.41	4.99E-130	-0.28	down	GSE122960	AT2	AT2	epithelial
**PPP1R15A**	-0.36	0.66	0.78	2.77E-122	-0.12	down	GSE135893	Ciliated	Ciliated	epithelial
**PPP1R15A**	-0.35	0.39	0.61	1.83E-80	-0.21	down	GSE135893	AT2	AT2	epithelial
**PPP1R15A**	-0.26	0.09	0.22	6.80E-04	-0.14	down	GSE128033	AT2	AT2	epithelial
**PPP1R15A**	0.25	0.35	0.13	2.10E-09	0.21	up	GSE128033	AT1_or_Club	AT1/club	epithelial
**ATF4**	0.33	0.85	0.75	2.84E-19	0.10	up	GSE128033	Endothelial	Endothelial	endothelial
**PPP1R15A**	-0.48	0.25	0.55	1.80E-16	-0.30	down	GSE122960	Endothelial	Endothelial	endothelial
**PPP1R15A**	-0.43	0.40	0.64	8.30E-11	-0.24	down	GSE135893	Lymphatic Endothelial Cells	Lymphatic	endothelial
**PPP1R15A**	-0.38	0.61	0.74	1.07E-57	-0.13	down	GSE135893	Endothelial Cells	Endothelial	endothelial
**PPP1R15A**	0.30	0.59	0.42	1.02E-26	0.16	up	GSE128033	Endothelial	Endothelial	endothelial
**PPP1R15A**	0.36	0.44	0.25	1.85E-07	0.20	up	GSE136831	VE_Capillary_B	Endothelial	endothelial

Table S2. Differential expression of PPP1R15A, DDIT3 and ATF4 across different lung cells in IPF versus control. Shown are the results that were significant with p_val_adj < 0.1. Avg_logFC; average log2 fold change, pct.1; percentage of cell in group 1 (IPF) expressing gene , pct.2; percentage of cells in group2 (Controls cells) expressing gene, p_val_adj; p-value adjusted for multiple testing. pct.diff ; difference in pct1 and pct.2, dir; direction of change, study; the GEO ID of the study used for the data, Cell (Author ID); The cell classification as defined in the study by the authors, Class; a uniform broad classification of the cell type across studies, Type; the cell type/class.
