# Supplementary material for: Sensitization of the UPR by loss of PPP1R15A promotes fibrosis and senescence in IPF
Source: Sci Rep. 2021 Nov 3;11:21584. doi: 10.1038/s41598-021-00769-7 (PMC8566588; doi:10.1038/s41598-021-00769-7)
Supplement: Supplementary file 2 — Supplementary Information. [file 41598_2021_769_MOESM2_ESM.docx]

**Supplemental Information**

**Supplemental Methods**

*TGFβ1 activation assay for western blot*

Fibroblasts (Lonza, cat. no. CC2512 lot no. 18TL057581) were seeded in 12 well plates at 1x10^5^ cells/well and allowed to adhere for 24 h. Next, cells were washed twice with 500 µl DPBS and starved for 24 h in serum free DMEM (Gibco, cat no 31966). Cells were stimulated for 24 h with TGFβ1 at the same final concentration (0.123 ng/ml) used to assess gene expression changes. Additionally, a higher concentration of TGFβ1 (10 ng/ml) was included and tunicamycin (0.05 µg/ml and 0.2 µg/ml; Sigma-Aldrich, cat no. SML1287) was included as a control of ER stress induction.

Cells were washed in PBS and lysed in 30 µl RIPA buffer (Sigma-Aldrich, cat. no. R0278) supplemented with Halt^TM^ protease and phosphatase inhibitor cocktail (Thermo Fisher Scientific, cat no. 78440). Lysates were quantified by BCA analysis (Thermo Fisher Scientific, cat. no. 23225), suspended in LDS sample buffer, denatured, run on an SDS-PAGE gel and transferred to a PVDF membrane (Bio Rad, cat. no. 1704157). A molecular weight ladder of SeeBlue Plus 2 (Invitrogen, cat. no. LC5925) mixed with MagicMark XP (Invitrogen, cat. no. LC5602) was included. The membrane was cut below the 50KDa marker and the membranes were blocked for 1 h at room temperature with 5% skimmed milk/PBSTween and incubated overnight at 4^O^C with, rabbit monoclonal anti-BiP (Cell Signalling Technology, cat. no. 3177S) or mouse monoclonal anti-CHOP (Cell Signalling Technology, cat. no. 2895S) diluted 1:1000 in blocking buffer. The blots were incubated with HRP-conjugated anti-rabbit IgG secondary antibody (for PPP1R15A and BiP; R&D Systems, cat. no. HAF008) or HRP-conjugated anti-mouse IgG (for CHOP; R&D Systems, cat. no. HAF007) and developed using a chemiluminescent system (BioRad, cat. no. 1705062). The blots were then stripped, blocked and incubated overnight at 4^O^C with either rabbit polyclonal anti-PPP1R15A (Proteintech, cat. no. 10449-1-AP) or rabbit polyclonal anti-GAPDH (Cell Signalling Technology, cat. no. CST2118S) diluted 1:1000 in blocking buffer. The blots were incubated with HRP-conjugated anti-rabbit IgG secondary antibody (R&D Systems, cat. no. HAF008) and developed using a chemiluminescent system (BioRad, cat. no. 1705062). All blots were imaged on a ChemiDoc MP (Bio-Rad) using ImageLab software.

*Capillary western for detection of peIF2a*

To determine the effect of TGFβ1 stimulation on phosphorylation of eIF2a, fibroblasts (Lonza, cat. no. CC2512 lot no. 19TL149590) were stimulated with TGFβ1 or tunicamycin and lysed and protein quantified as described above. Samples were loaded on a capillary western machine (WES, ProteinSimple) and run according to the manufacturer’s instructions. Lanes were loaded with anti-eIF2a (Cell Signalling Technology, cat. no. 5324; diluted 1:10,000) or anti-p-eIF2a (Cell Signalling Technology, cat. no. 3398; diluted 1:10). All lanes were loaded with anti-vinculin (Abcam, cat. no. 129002; diluted 1:1000) as a loading control. Samples were analysed using Compass for SW software.

**Supplemental Figures**

**
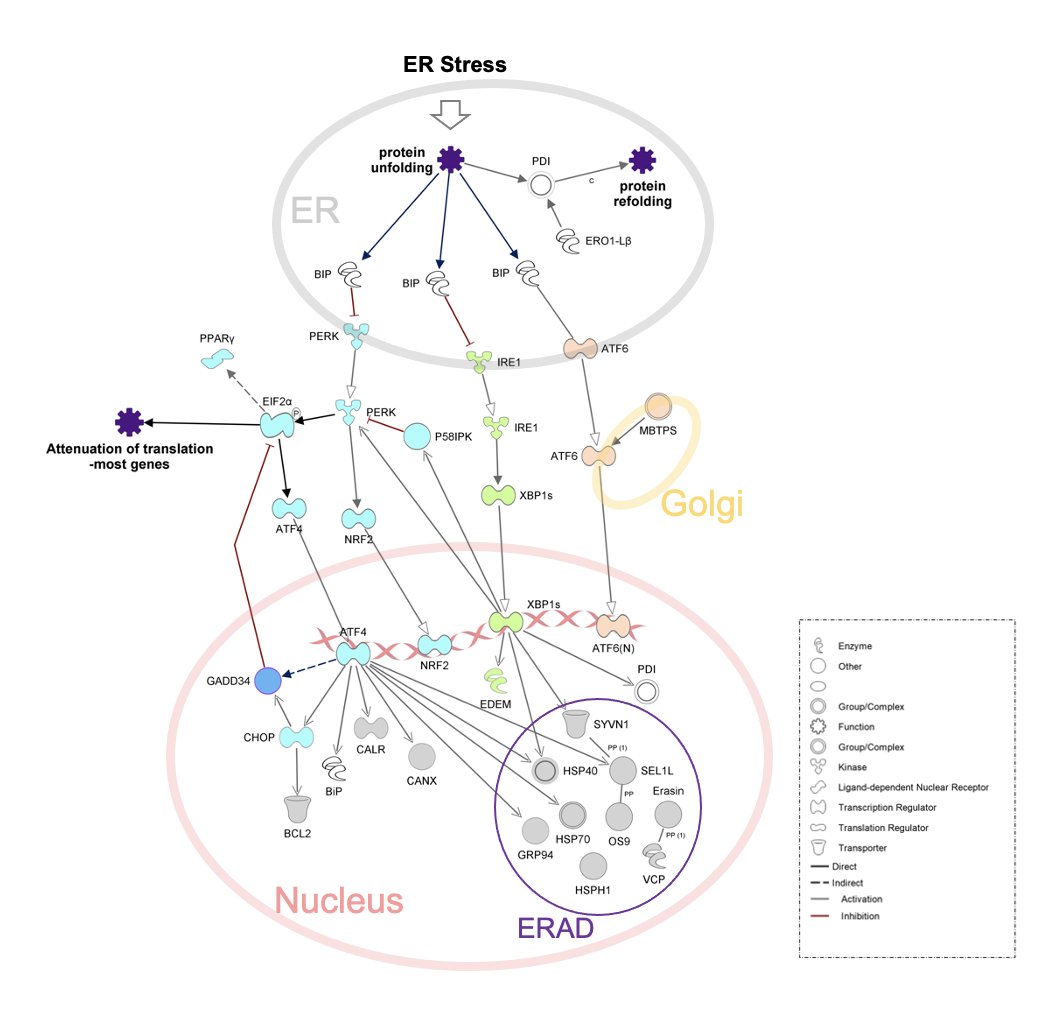
**

**Figure S1. Key UPR pathway genes.** A schematic illustrating the 3 UPR pathways; PERK (Blue), IRE1(Green) and ATF6 (Orange) and the genes associated or regulated by them based on Ingenuity Pathway Analysis (IPA) interactions. Relationship indicated with open arrow are translocation of molecule or it’s function; relationships indicated by filled arrowhead are functional interactions and gene expression regulation is illustrated with line arrows. The network figure was generated through the use of Path Designer from Ingenuity Pathway Analysis ([https://digitalinsights.qiagen.com/products-overview/discovery-insights-portfolio/analysis-and-visualization/qiagen-ipa/](https://gbr01.safelinks.protection.outlook.com/?url=https%3A%2F%2Fdigitalinsights.qiagen.com%2Fproducts-overview%2Fdiscovery-insights-portfolio%2Fanalysis-and-visualization%2Fqiagen-ipa%2F&data=04%7C01%7Cl.murray%40mirobio.com%7C203e80f222f046dbe78c08d9694a5edb%7Cffa065f885a444f9847f2c3302345733%7C0%7C0%7C637656590419809126%7CUnknown%7CTWFpbGZsb3d8eyJWIjoiMC4wLjAwMDAiLCJQIjoiV2luMzIiLCJBTiI6Ik1haWwiLCJXVCI6Mn0%3D%7C1000&sdata=qfmf66s4vZpjhZ%2Fy2vbwnIhPGaYlhEi3Uoo9kosNp38%3D&reserved=0)).

**
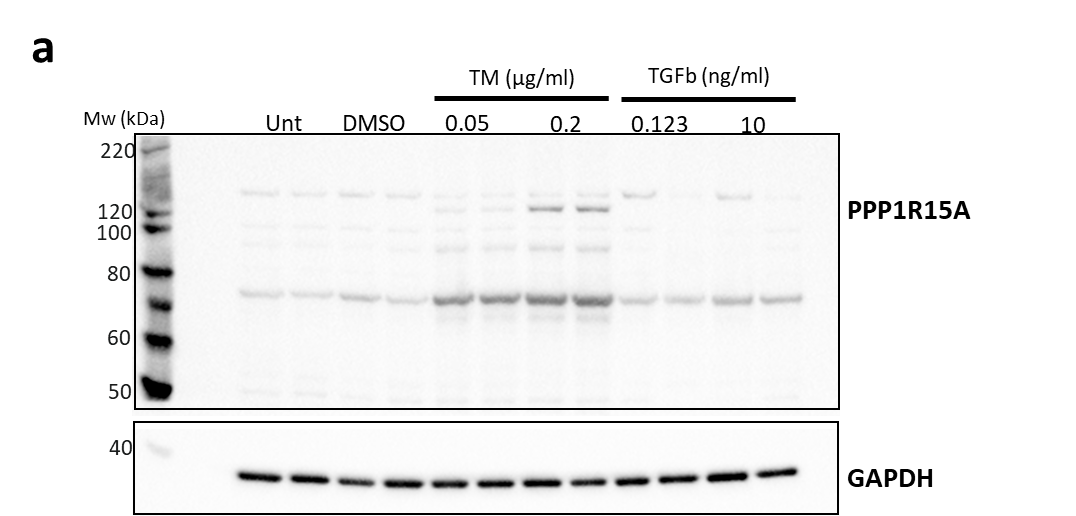
**

**
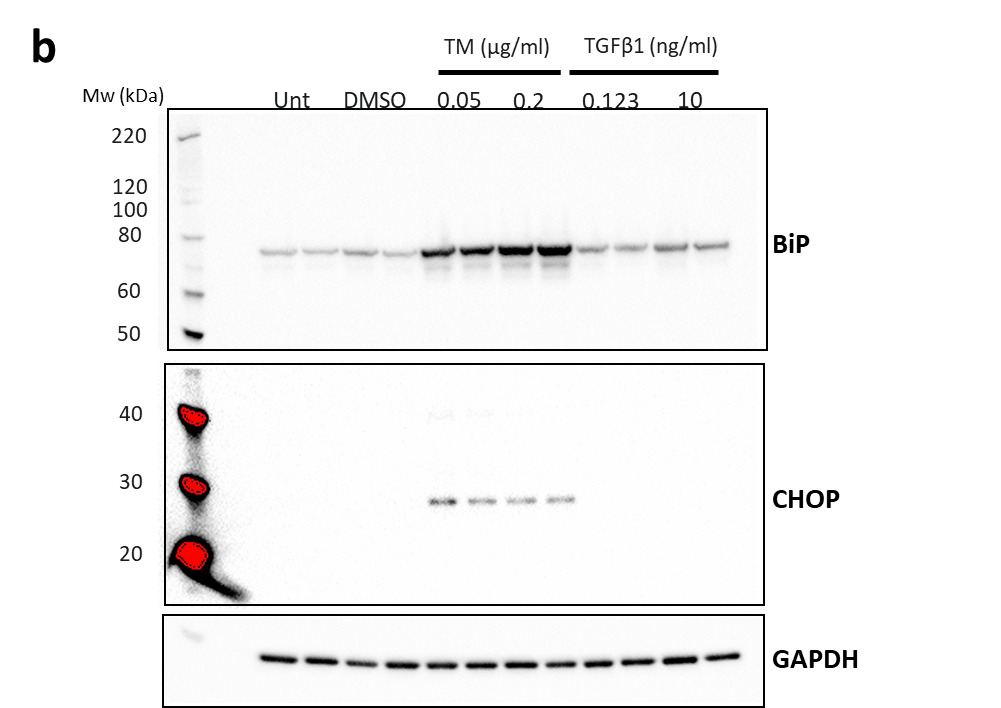
**

**
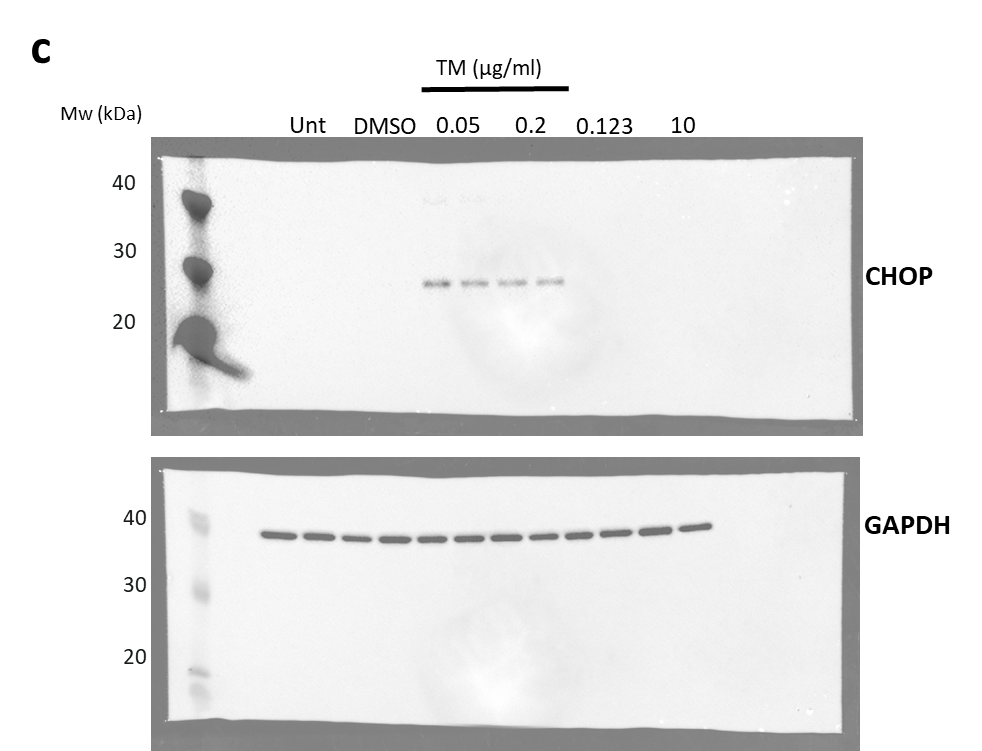
**


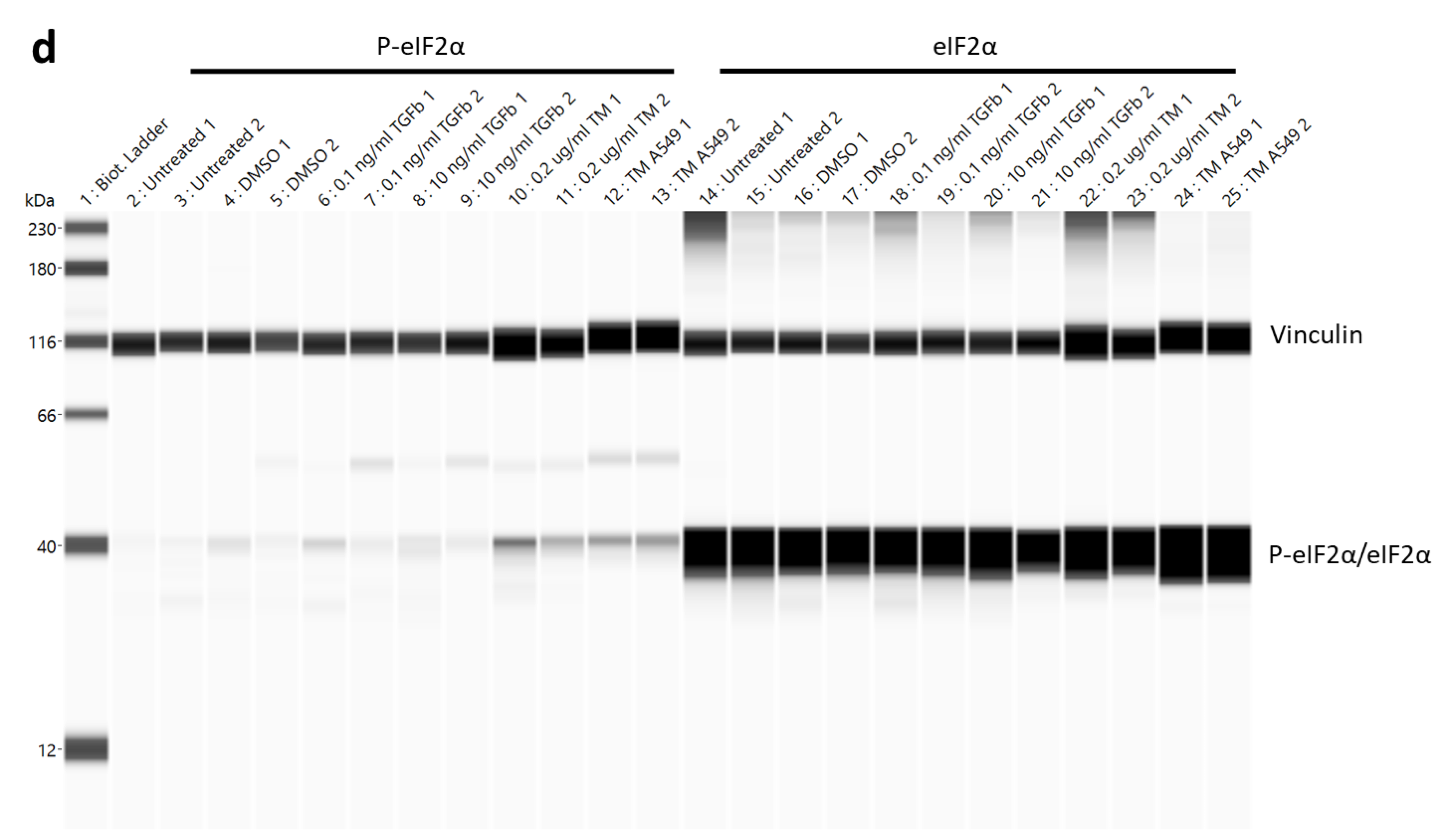


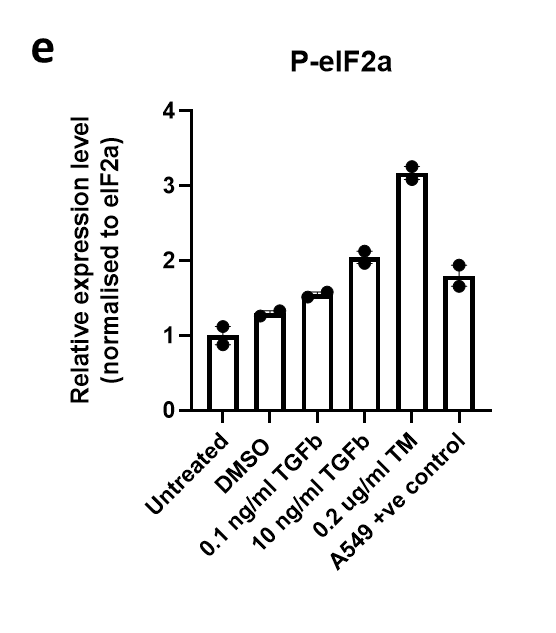


**Figure S2. Western Blot analysis of PPP1R15A, CHOP, BiP and pEIF2a in Lung Fibroblasts.**

Western blot analysis of PPP1R15A expression (and GAPDH loading control) (**a**) and BiP and CHOP (and GAPDH loading control) (**b**) in human lung fibroblasts treated for 24 h with TGFβ1 (0.123 ng/ml or 10 ng/ml) or tunicamycin (0.05 µg/ml or 0.2 µg/ml; TM). DMSO included as vehicle control for tunicamycin. Unt; untreated. Duplicate treatments shown. Blots for GAPDH were cropped. Multichannel images (chemiluminescent and colorimetric) depicting full blots for CHOP and GAPDH **(c).** Capillary western for p-eIF2a and total eIF2a (vinculin loading control) **(d).** Densitometry of peIF2a normalised to total eIF2a **(e).**


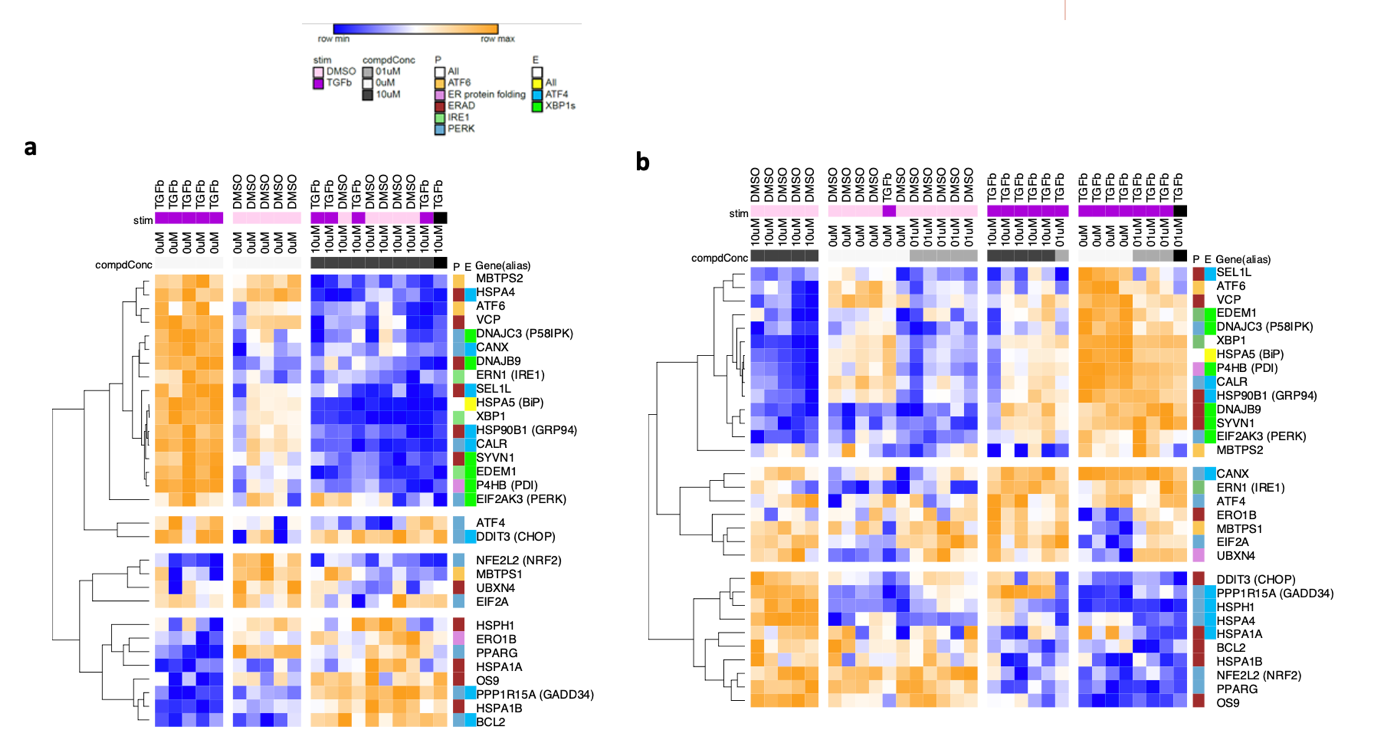


**Figure S3. Modulation of TGFβ1 altered UPR pathway genes by compounds.** Heatmap of gene expression (log2(TPM)) of the UPR genes from Table S1 for IPF fibroblasts (5 donors, 3-5 replicates) treated with TGFβ1 (0.123ng/ml) for 24h and additionally with nintedanib (**a**) or AZD8055 (**b**) at concentrations shown. The genes are further colour coded into pathway (P) and expression regulators (E) according to Table S1. (**a**) 10 μM nintedanib reverses the induction of expression caused by TGFβ1 for a large set of the UPR genes (top panel) including the majority of genes downstream of ATF4 and all the gens downstream of XBP1s. For another set of genes, including PPP1R15A, 10 μM nintedanib reversed the repression of TGFβ1 (bottom panel). (**b**) The mTOR inhibitor AZD8055 is also able to reverses the induction of expression caused by TGFβ1 for a similar set of UPR genes to nintedanib (top panel). Similarly AZD8055 reversed the repression of TGFβ1 (bottom panel) for another set of genes including PPP1R15A.


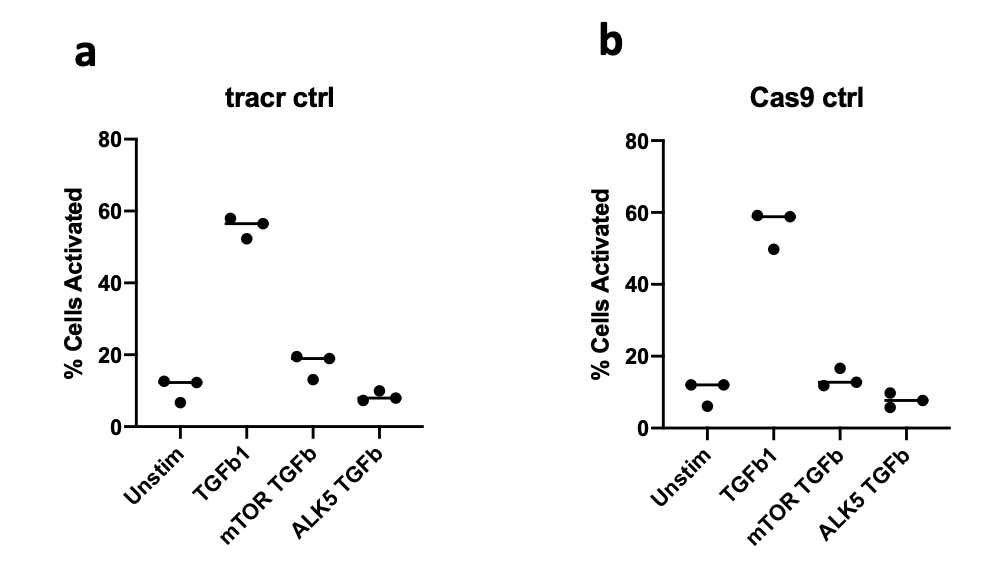


**Figure S4. No effect of CRISPR controls on TGFβ-induced fibroblast activation.** TGFβ-induced myofibroblast activation was quantified in normal human primary fibroblasts electroporated with Tracr and Cas9 protein only controls. Cells in starvation conditions were incubated with positive control compounds (mTOR inhibitor and ALK5 inhibitor) (3 µM) for 1 hr before treatment with TGFβ (0.125 ng/ml) for 24 h.  Unstimulated cells were treated with 0.1% (v/v) DMSO for normalisation. Myofibroblast activation was measured by immunofluorescence of αSMA and represented as percent cell activation, based on Columbus linear classifier algorithms using intensity, texture and morphology features.


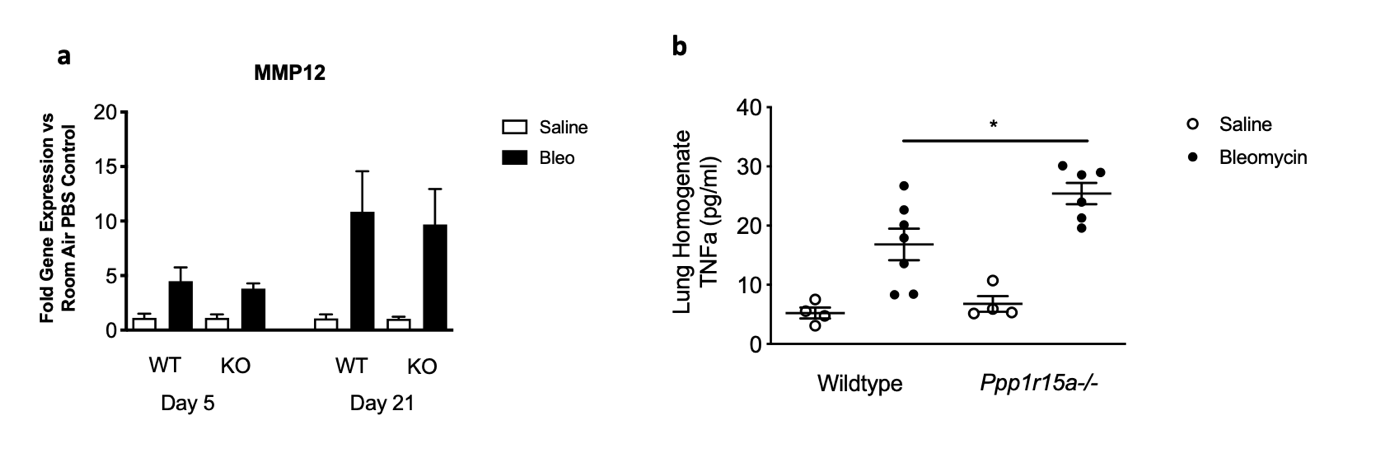


**Figure S5.** **Loss of PPP1R15A has no impact on bleomycin-induced MMP12 but does impact bleomycin-induced TNFα protein levels** (**a**) Whole lung gene expression of *Mmp12* at Day 5 and Day 21 after intratracheal bleomycin or saline control in wildtype (WT) and *Ppp1r15a-/-* (KO) mice. Bars represent mean ± s.e.m. of n= 4-7 mice per group. Gene expression quantified using RT-PCR. (**b**) Whole lung TNFα protein levels were measured using MSD technology in lung homogenates, at Day 21 post-bleomycin or saline challenge in wildtype or *Ppp1r15a-/-* mice. Dots represent individual animals, bars represent mean ± s.e.m. **P*<0.05


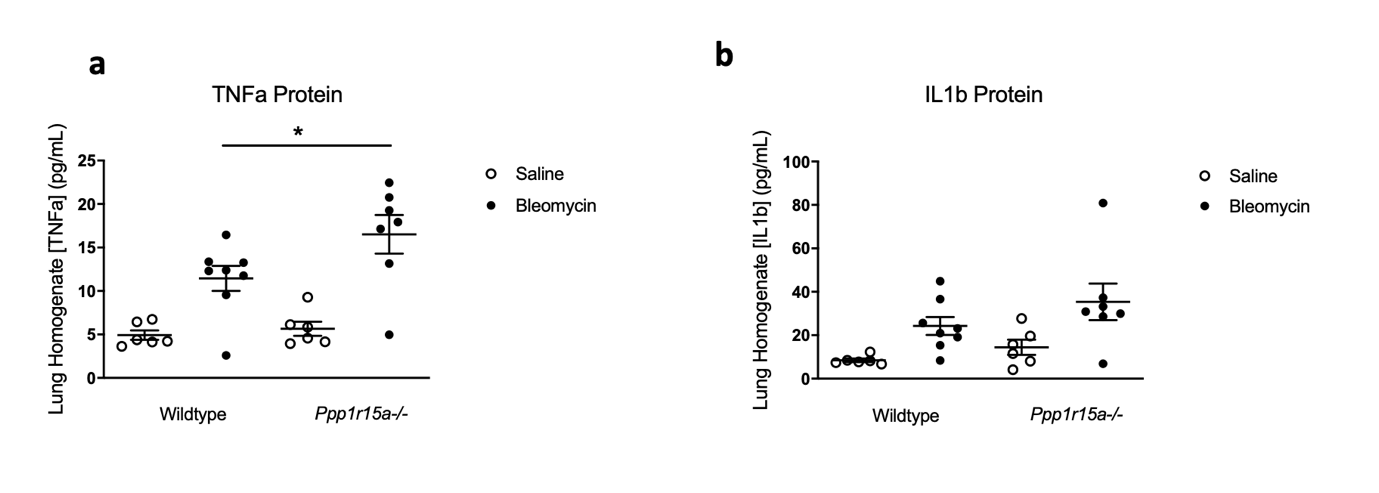


**Figure S6.** **PPP1R15A gene deficient animals had reduced bleomycin-induced lung inflammation at Day 5.** Whole lung TNFα (**a**) and IL1β (**b**) protein levels were measured using MSD technology in lung homogenates, at Day 5 post-bleomycin or saline challenge in wildtype or *Ppp1r15a-/-* mice. Dots represent individual animals, bars represent mean ± s.e.m. **P*<0.05


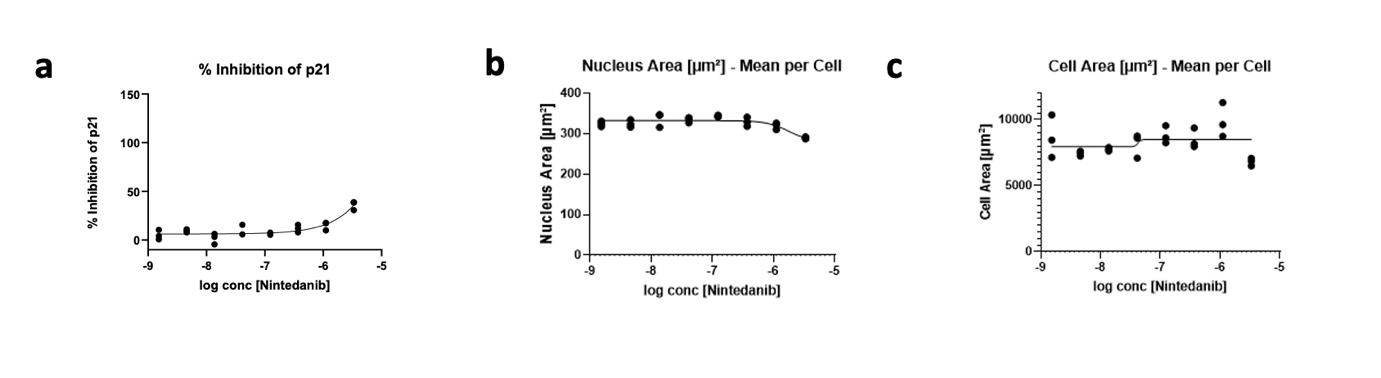


**Figure S7. Nintedanib has no effect on etoposide-induced fibroblast senescence.** IPF fibroblasts were cultured in the presence of etoposide (3 µM) and with the indicated nintedanib concentrations for 72hrs, after which the cells were stained with an anti-p21 antibody as well as the cell and nucleus stains CellMask and Hoechst. The cells were imaged and the p21 inhibition percentage (**a**), nucleus area (**b**) and cell area (**c**) with nintedanib was determined using image analysis. Representative data for n=1 IPF donor cell lines with each dot representing technical replicates.


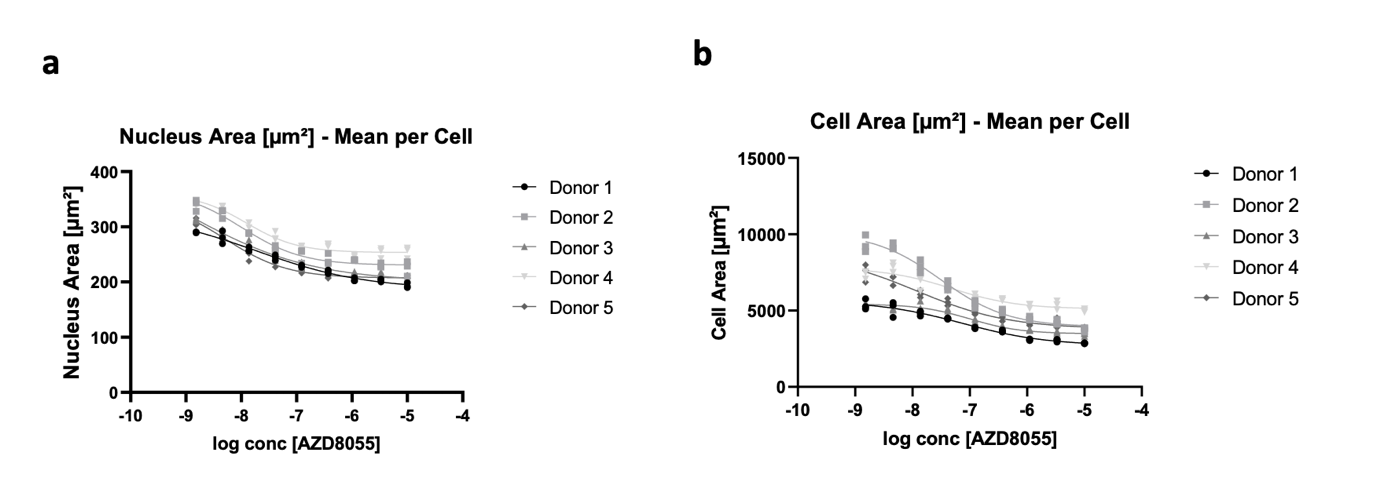


**Figure S8. Etoposide-induced senescence in IPF lung fibroblasts is inhibited by the mTOR inhibitor AZD8055.** IPF fibroblasts were cultured in the presence of etoposide (3 µM) and with the indicated compound concentrations for 72hrs, after which the cell and nucleus areas quantified using CellMask and Hoechst staining and size quantified using image analysis. n=5 IPF donor cell lines.
